# Identification of phosphorylated tau protein interactors in progressive supranuclear palsy (PSP) reveals networks involved in protein degradation, stress response, cytoskeletal dynamics, metabolic processes, and neurotransmission

**DOI:** 10.1111/jnc.15796

**Published:** 2023-03-21

**Authors:** Rowan A. W. Radford, Stephanie L. Rayner, Paulina Szwaja, Marco Morsch, Flora Cheng, Tianyi Zhu, Jocelyn Widagdo, Victor Anggono, Dean L. Pountney, Roger Chung, Albert Lee

**Affiliations:** ^1^ Centre for Motor Neuron Disease Research Macquarie Medical School Faculty of Medicine, Health and Human Sciences Macquarie University New South Wales North Ryde Australia; ^2^ Clem Jones Centre for Ageing Dementia Research, Queensland Brain Institute The University of Queensland Queensland Brisbane Australia; ^3^ School of Pharmacy and Medical Sciences Griffith University Queensland Gold Coast Australia

**Keywords:** Biotinylation, mass spectrometry, neuropathology, progressive supranuclear palsy, tau, Tauopathy

## Abstract

Progressive supranuclear palsy (PSP) is a late‐onset neurodegenerative disease defined pathologically by the presence of insoluble phosphorylated‐Tau (p‐Tau) in neurons and glia. Identifying co‐aggregating proteins within p‐Tau inclusions may reveal important insights into processes affected by the aggregation of Tau. We used a proteomic approach, which combines antibody‐mediated biotinylation and mass spectrometry (MS) to identify proteins proximal to p‐Tau in PSP. Using this proof‐of‐concept workflow for identifying interacting proteins of interest, we characterized proteins proximal to p‐Tau in PSP cases, identifying >84% of previously identified interaction partners of Tau and known modifiers of Tau aggregation, while 19 novel proteins not previously found associated with Tau were identified. Furthermore, our data also identified confidently assigned phosphorylation sites that have been previously reported on p‐Tau. Additionally, using ingenuity pathway analysis (IPA) and human RNA‐seq datasets, we identified proteins previously associated with neurological disorders and pathways involved in protein degradation, stress responses, cytoskeletal dynamics, metabolism, and neurotransmission. Together, our study demonstrates the utility of biotinylation by antibody recognition (BAR) approach to answer a fundamental question to rapidly identify proteins in proximity to p‐Tau from post‐mortem tissue. The application of this workflow opens up the opportunity to identify novel protein targets to give us insight into the biological process at the onset and progression of tauopathies.

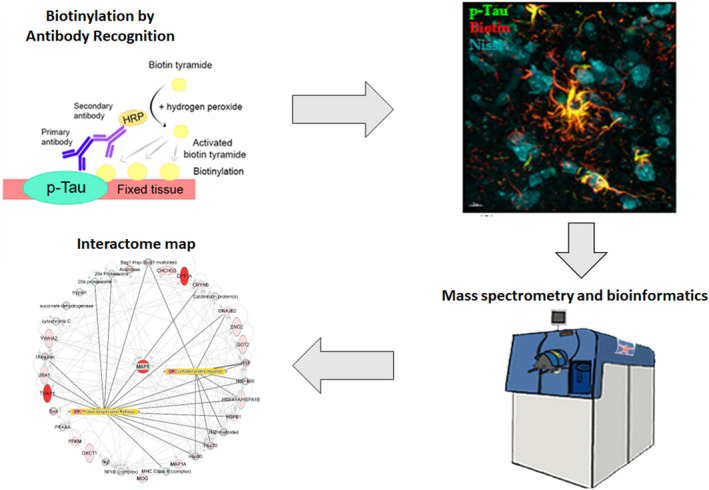

## Graphical abstract

Identifying co‐aggregating proteins within p‐Tau inclusions reveals important insights into processes affected by the aggregation of Tau. We used a proteomic approach, which combines antibody‐mediated biotinylation and mass spectrometry to identify proteins proximal to p‐Tau in cases of progressive supranuclear palsy. Bioinformatics tools revealed that the proteins identified within a small radius of p‐Tau were associated with neurological disorders and pathways involved in protein degradation, stress responses, cytoskeletal dynamics, metabolism, and neurotransmission. Ultimately, this workflow represents a method to rapidly characterize aggresomes that can be applied to multiple neurodegenerative disorders.

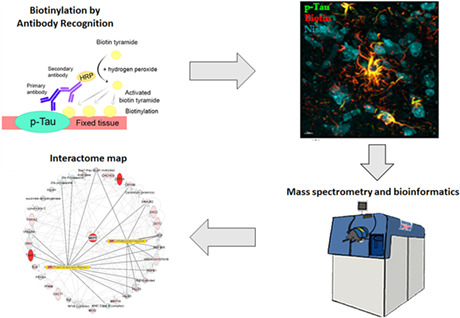

**Abbreviations**
4R: four‐repeat
AD: Alzheimer's disease
AmBic: ammonium bicarbonate
AGC: automatic gain control
BAR: biotinylation by antibody recognition
BCA: bicinchoninic acid assay
Bis‐Tris: 1,3‐bis(tris(hydroxymethyl)methylamino)propane
CB: coiled body
CBD: corticobasal degeneration
DTT: dithiothreitol
FA: formic acid
FT‐MS: fourier transform‐mass spectrometry
FDR: false discovery rate
GS: goat serum
GWAS: genome‐wide association study
HCD: higher energy collision dissociation
HRP: horseradish peroxidase
IAA: iodoacetamide
IHC: immunohistochemistry
IP: immunoprecipitation
IPA: ingenuity pathway analysis
LC‐MS/MS: liquid chromatography‐mass spectrometry
MS: mass spectrometry
NFT: neurofibrillary tangle
OPCs: oligodendrocyte precursor cells
PBS: phosphate‐buffered saline
PBST: phosphate‐buffered saline containing Tween‐20
PSM: peptide‐spectrum match
PiD: pick's disease
PMI: post‐mortem interval
PSP: progressive supranuclear palsy
RRID: Research Resource Identifier (see scicrunch.org)
RT: room temperature
SDS: sodium dodecyl sulfate
SDS‐PAGE: sodium dodecyl sulfate‐polyacrylamide gel electrophoresis
TA: Tufted Astrocyte
TBS: tris‐buffered saline

## 1 INTRODUCTION

Progressive supranuclear palsy (PSP) is a late‐onset, fatal neurodegenerative syndrome with heterogeneous clinical manifestations (Höglinger et al., 2017). Because of this heterogeneous phenotype, definitive diagnosis of PSP can only be confirmed at autopsy by the pathological presentation of neurodegeneration and specific hyperphosphorylated, four‐repeat (4R) Tau in neurons, and glia in anatomical regions corresponding to primary clinical symptoms (Kovacs et al., 2020). Because of the presence of pathological Tau, PSP is classified as a Tauopathy along with other neurodegenerative conditions such as corticobasal degeneration (CBD), Pick's disease (PiD), and Alzheimer's disease (AD) (Kovacs, 2015). However, despite the common Tau neuropathology, numerous studies have shown that Tau is processed distinctly in each of these conditions, which is reflected in differences in inclusion morphology, biochemical profile, and ultrastructure among the Tauopathies including PSP (Arakhamia et al., 2020; Falcon et al., 2019; Falcon, Zhang, Murzin, et al., 2018; Falcon, Zhang, Schweighauser, et al., 2018; Shi et al., 2021; Taniguchi‐Watanabe et al., 2016). This is also supported by comparative interactome studies of human and rodent tau protein networks that show distinct differences in their aggresome profiles (Kavanagh et al., 2022), which potentially makes studying cellular mechanisms of the human condition difficult to recapitulate in rodent models.

Further highlighting the causative role of Tau in disease pathogenesis, mutations to the gene encoding Tau (*MAPT*) can result in clinical and neuropathological PSP (Chen et al., 2019; Forrest et al., 2018; Fujioka et al., 2015; Im et al., 2015). Moreover, genome‐wide association studies have identified variants in the *MAPT* locus in conferring the most significant genetic risk associated with developing PSP (Höglinger et al., 2011). Collectively, neuropathology, biochemistry, and genetics strongly link altered biochemical forms of Tau to PSP pathogenesis. Several studies have performed biochemical extraction of phosphorylated Tau (p‐Tau) from PSP tissue to identify the specific features of insoluble pathogenic Tau. These studies have identified distinct structural changes in Tau such as aggregation, fibrillization, and post‐translational modifications of which phosphorylation is most prominent (Taniguchi‐Watanabe et al., 2016; Wray et al., 2008). Tau is ordinarily a highly phosphorylated protein, and disease‐specific phosphosites have been identified (Arendt et al., 2016).

In addition to post‐translational modifications of Tau, identifying proteins in proximity to Tau has provided further insights into disease progression and pathogenesis that are common in Tauopathies. For example, several nuclear speckle proteins have been found to co‐localize with p‐Tau inclusions in tauopathies, including AD, frontotemporal dementia, and CBD, providing links between Tauopathies and defects in RNA processing (Lester et al., 2021). A classical approach used to identify Tau inclusion components from post‐mortem brain tissue involves enriching the detergent‐soluble aggregates from post‐mortem brain tissue using biochemical fractionation methods, after which the detergent‐insoluble proteins are detected using mass spectrometry (MS). While this approach has provided valuable insight into the detergent‐insoluble aggregate composition, technical limitations include a high degree of non‐specific detection and loss of proteins through harsh biochemical processing. Another classical method is to co‐immunoprecipitate Tau with interaction partners from the soluble and sarkosyl‐insoluble fractions of post‐mortem tissue. However, this relies on the stable interaction being maintained between Tau and interaction partners through the disruptive extraction process (Julien et al., 2012). Alternate techniques such as immunohistochemistry (IHC), biotinylation (Prikas et al., 2022), and proximity‐ligation detection methods provide precise in situ labeling of individual protein components but are limited by antibody combination limits (Duraiyan et al., 2012; Söderberg et al., 2008).

Biotinylation by antibody recognition (BAR) is a recently described proximity‐ligation method that is particularly well‐suited for analyzing formalin‐fixed, post‐mortem tissue from patients (Bar et al., 2018). BAR uses a primary antibody that recognizes the target of interest in fixed samples. A secondary antibody conjugated to horseradish peroxidase (HRP) recognizes the primary antibody and, with the addition of biotin phenol and hydrogen peroxide, facilitates the rapid conjugation of biotin onto proteins within the vicinity of the antibody complex. In contrast to classical biochemical extractions, BAR enables labeling of the endogenous, aggregating proteins before tissue homogenization. Therefore, even if the aggregate is disrupted during the subsequent extraction process, the BAR‐labeled proteins that have been separated from the aggregate can still be identified. Thus, this method significantly enhances the likelihood of identifying proteins within protein aggregates, including low‐abundance proteins.

Proximity‐ligation methods such as BAR, proximity‐dependent biotin identification (BioID), and an engineered ascorbate peroxidase method (APEX) are all based on the covalent attachment of biotin to proteins in proximity to a protein of interest via enzymatic activity after which the biotinylated proteins are isolated using streptavidin‐conjugated beads and identified by MS (Hedl et al., 2019; Lobingier et al., 2017; Roux et al., 2012). Notably, these methods provide additional advantages in studying insoluble protein inclusions because of the strong interaction between biotin and streptavidin, which can withstand harsh extraction conditions, including buffers containing ionic detergents and chaotropic reagents such as sodium dodecyl sulfate (SDS) and urea. However, the fundamental constraints of other proximity‐ligation methods, such as BioID and APEX, are that they must be performed in living cells and require genetic alteration or transfection. As a result, BAR represents a novel method for analyzing protein–protein interactions within patient post‐mortem tissue samples.

In order to answer one of the most fundamental questions in tau pathology and proteomics, as a proof‐of‐concept, we have applied BAR followed by MS to identify proteins that are proximal to phosphorylated‐Tau (p‐Tau) inclusions in post‐mortem cases of PSP. We demonstrate the utility of this method to rapidly identify proteins beyond Tau that aggregate in the neurons and glia of PSP patients. Notably, we were able to identify previously reported Tau phosphorylation sites as well as p‐Tau interaction partners. Furthermore, analysis of the p‐Tau‐aggregate interactome also revealed multiple proteins in proximity to p‐Tau, which may reveal further insights into the molecular pathways that may be affected in PSP.

## 2 MATERIALS AND METHODS

### 2.1 Post‐mortem tissue

As a proof‐of‐concept to apply the BAR experimental approach to characterize the p‐Tau aggresome, we have used formalin‐fixed post‐mortem tissue blocks limited to 4 cases with PSP (Table 1) were obtained from the South Australian Brain Bank under ethics approvals MSC/16/11/HREC and HREA 5201600387. An exclusion post‐mortem interval (PMI) criteria of >24 were used to select tissue samples that had minimal amounts of protein degradation (Blair et al., 2016). The blocked motor cortex of 4 PSP patients was cut to fit within a 12 mm^2^ template before being serially sectioned on a Leica VTS1200 vibratome at 50–85 μm into 24‐well plates. Individual sections were weighed before subsequent immunohistochemistry, imaging, or proteomics. As this was a proof‐of‐concept study to identify p‐Tau proximal proteins using BAR, blinding and sample power calculations were not performed for this study. Other proof‐of‐concept studies that have developed new techniques for tissue proteomics have used similar small sample sizes (Blair et al., 2016; Drummond et al., 2015).

**TABLE 1:** Details of patient post‐mortem tissue used for study.

Case	Gender	Age (yrs)	PMI (hrs)	Duration (months)	Clinical diagnosis	Neuropathological diagnosis
0082	F	73	4	60	PSP* (suspected CBD)	PSP
0095	M	73	4.5	60	PSP	PSP‐RS
0122	M	79	16	48	PSP	PSP‐RS
0136	F	80	17	72	PSP	PSP‐RS
*N* = 4	2:2 (M:F)	76^3.8^ (avg.^SD^)	10.4^7.1^ (avg.^SD^)	60^9.8^ (avg.^SD^)		PSP‐RS = 3 PSP = 1

Abbreviations: avg., average; CBD, corticobasal degeneration; F, female; M, male; PMI, post‐mortem interval; PSP, progressive supranuclear palsy; RS, Richardson's syndrome; SD, standard deviation.

### 2.2 Immunohistochemistry and proximity biotinylation

Vibratome (Leica Biosystems, RRID:SCR_018453) sections were washed with tris‐buffered saline (TBS) before endogenous peroxidases were quenched by incubating in 1% H_2_O_2_ (Invitrogen, Catalog number: B40911) solution in TBS for 1 hr at room temperature (RT). Sections were then washed and blocked for 2 hrs at RT in 10% (v/v) goat serum (GS, Invitrogen, Catalog number: B40911) + 0.3 M glycine (Sigma, Catalog number: 50046) in TBS. Primary antibody recognizing p‐Tau (clone AT8, MN1020 Invitrogen, RRID: AB_223647) was diluted 1:800 in 1% GS in TBS, while negative control sections with primary antibodies omitted were incubated on sections for two nights at 4°C. Sections were washed before goat‐conjugated Superboost™ HRP (Invitrogen, Catalog number: B40961) was added to 1% GS and incubated overnight at 4°C. The next day, sections were washed and then incubated with biotin‐XX‐tyramide reagent (Invitrogen, Catalog number: B40951) for 45 min at RT. The biotinylation reaction was conducted for 60 s with 0.03% H_2_O_2_ before the solution was removed. The stop solution was immediately added before being washed.

To assess the biotinylation, fluorescently conjugated streptavidin (Atto‐Tec, Catalog number: AD647N61) and secondary directed against the primary antibody were added overnight at 4°C, with all subsequent steps being performed in the dark. The following day, sections were washed and then counterstained with either DAPI (Invitrogen Catalog number: D1306) or NeuroTrace™ Blue (Invitrogen, Catalog number: N21479) for 90 min in TBS before being washed, mounted with Prolong™ Glass Antifade Mountant (Thermo, Catalog number: P36984), and a coverslip applied. Finally, the sections were imaged using confocal microscopy (Leica Microsystems SP5x with resonant scanner RRID:SCR_018714).

### 2.3 Biotin‐streptavidin pull‐downs

In order to isolate biotinylated proteins, biotinylated tissue was first homogenized using a hand‐held douncer in BAR lysis buffer (1% SDS (Sigma, Catalog number: L3771), 1% sodium deoxycholate (Merck, Catalog number: D6750)). After homogenization, tissue was probe‐sonicated 10 times using a Sonic Ruptor 250 at 50% power and pulser settings set to 30%. Samples were then heated at 95°C for 1 h and then further heated to 60°C for 2 h to reverse cross‐link the fixed tissue. After reverse cross‐linking, samples were cooled to 4°C before appropriate amounts of protease and phosphatase inhibitor cocktail (Roche, Catalog numbers: 04693159001 and 04906837001) were added to samples. The resulting lysates were centrifuged at 1000 *g* for 5 min at 4°C.

Proteins within the clarified lysate were quantified using the Pierce BCA protein assay kit (Thermo, Catalog number: 23225). Equal amounts of protein were aliquoted before 50 μL of pre‐washed streptavidin‐coated magnetic sepharose beads (GE) were added, and samples were left to rotate overnight at 4°C. A magnetic rack was then used to isolate magnetic beads and associated biotinylated proteins. To reduce non‐specific binding, magnetic beads were washed twice with BAR lysis buffer, then twice with RIPA lysis buffer (50 mM Tris–HCl, pH 7.5, 150 mM NaCl, 1% NP‐40, 1 mM EDTA, 1 mM EGTA, 0.1% SDS, 0.5% sodium deoxycholate, pH 7.4), once with 1 M KCl, 1 M Na_2_CO_3_, 2 M urea (in 10 mM Tris–HCl, pH 8.0) and finally twice with RIPA buffer and BAR lysis buffer.

### 2.4 Immunoblotting

Equal amounts of protein were separated on a 4–15% gradient SDS‐PAGE gel (Bio‐Rad, Catalog number: 4561085). The separated proteins were transferred onto a nitrocellulose membrane using a Trans‐Blot Turbo Transfer System (Bio‐Rad). The resulting membranes were blocked with 3% bovine serum albumin (BSA, Sigma, Catalog number: A9418) in PBS containing 0.05% v/v Tween (3% (w/v) BSA in PBS‐T) for 1 h. Streptavidin conjugated to a fluorophore (IRDye 800CW streptavidin, LI‐COR, catalog number:926‐32 230) was diluted 1:1000 in 3% (w/v) BSA in PBS‐T before being incubated with the blocked membrane for 30 min. The membrane was then washed three times in PBS‐T and imaged using the Li‐Cor Odyssey CLx Imaging System at the appropriate wavelengths. Images were analyzed using Image Studio Lite Software (RRID: SCR_002285).

### 2.5 On‐bead trypsin digest

Before trypsin digests were conducted, excess detergent was washed from the beads by resuspending the beads five times in 20 mM ammonium bicarbonate (AmBic, Sigma, Catalog number: A6141). Washed beads were incubated with 10 mM Dithiothreitol (DTT, Sigma, Catalog number: D9779) in 20 mM AmBic for 1 h at 60°C. Samples were then alkylated using 15 mM of iodoacetamide (IAA, Sigma, Catalog number: I6125) in 20 mM AmBic for 1 h at room temperature in the dark. For trypsin digestion, proteins were incubated with 5 μg/mL of sequencing grade trypsin (Thermo, Catalog number: 90057) overnight at 37°C whilst shaking. The resulting peptides were lyophilized using a SpeedVac. For desalting, peptides were resuspended in 0.1% formic acid (FA, Thermo, Catalog number: 85178) and desalted using pre‐washed and equilibrated C18 OMIX tips (Agilent, Part No: A57003100K). Once desalted, samples were again lyophilized using a SpeedVac before being stored at −80°C until MS analysis.

### 2.6 Reverse phase C_18_ liquid chromatography‐mass spectrometry

Before MS analysis, lyophilized peptides were resuspended in 0.1% FA and bath sonicated for 15 min. Samples were then centrifuged at 14 000 *g* for 15 min at 4°C, and supernatants were used for analysis by liquid chromatography‐mass spectrometry (LC–MS/MS). To conduct analyses, peptides were separated on an Ultimate 3000 nanoLC (Thermo Fisher Scientific) containing an Acclaim PepMap RSLC column (particle size 2 μm, diameter 0.075 mm and length 150 mm, Thermo), employing a 120‐minute gradient (2–80% v/v acetonitrile, 0.1% v/v FA) and 300 nL/min flow rate.

The eluted peptides were ionized into the Q Exactive™ Plus mass spectrometer (Thermo). The electrospray source was fitted with an emitter tip (10 μm, New Objective) and was maintained at 1.5 kV electrospray voltage. The capillary temperature was set to 250°C. A data‐dependent “Top 15” method was used to select precursor ions prior to MS/MS fragmentation, while an FT‐FT acquisition mode was used with HCD fragmentation. FT‐MS analysis was conducted at 70 000 resolution with an automatic gain control (AGC) target of 1 × 10^6^ ions in full MS, whilst MS/MS scans were conducted at 35 000 with an AGC target of 2 × 10^5^ ions. Maximum injection times were 120 ms. The ion selection threshold used to trigger MS/MS fragmentation was set to 25 000 counts. For HCD fragmentation, an isolation width of 1.4 m/z was chosen. Normalized collision energy of 27 was used.

The resulting raw files were searched using Proteome Discoverer 2.4 software (Thermo, RRID: SCR_014477), which used the Sequest search algorithm. Here, the *Homo sapiens* Uniprot FASTA database 9606 was used. For peptide identifications, the settings accounted for a 20‐ppm precursor ion FT‐MS tolerance as well as a 0.1 Da MS/MS fragment ion tolerance for HCD fragmentation. For static modifications, the program was set to search for carbamidomethylation on cysteine. For dynamic modifications on the protein terminus, the program was set to Acetyl (N‐Terminal). The program was set to oxidation on methionine and phosphorylation on serine, threonine, and tyrosine for dynamic modifications. Finally, trypsin was assigned as the enzyme, allowing for up to three missed cleavages.

### 2.7 Statistical analysis of protein identifications

The MS data were further processed using a Label‐Free Quantitation workflow featuring the peptide spectrum match (PSM) Minora Feature node. This made use of the percolator node, enabling an estimation of false discovery rates (FDR) at the protein and PSM levels. Protein identifications were validated employing a q‐value of 0.01. Label‐free quantitation (LFQ) using intensity‐based quantification was carried out. LFQ was carried out according to default parameter settings in the Proteome Discoverer 2.4 Software (Thermo). Briefly, peptide spectral matches (PSM) were filtered using a maximum delta Cn of 0.05, rank of 0, and delta mass of 0 ppm. PSMs and peptides were, respectively, validated using a strict FDR for PSMs of 0.01 and 0.05 for a relaxed FDR. Peptides shorter than 6 amino acids were filtered out. PSMs were chromatographically aligned for each input file in a sample set with a mass tolerance of 10 ppm and a maximum retention time (RT) shift of 10 min. Peptide groups used for protein quantification were analyzed using the default parameters which set a peptide as unique if it is included in only one protein group. The quantification was processed using unique and razor peptides (peptides shared among multiple proteins group or proteins) with the precursor abundance based on the intensity. Protein abundance was calculated as a sum of the individual peptide group abundances, and the ratio was based on pairwise ratio using a geometric median of the peptide group ratios. An ANOVA test was used for the hypothesis test and uses the background population of ratios for all peptides and proteins to determine whether any given single peptide or protein is significantly changing relative to that background (Proteome Discoverer User Guide Software Version 2.4, Thermo).

### 2.8 Bioinformatics analysis

Ingenuity Pathway Analysis (IPA) (Qiagen, RRID: SCR_008653) was used to analyze the MS results. “Diseases and Disorders” function was employed to create pathways and interaction for “Neurological Disease” and “Molecular and Cellular Functions” if they contained 10 or more proteins found associated with p‐Tau from PSP samples were present. Canonical pathway analysis was employed to find pathways for multiple proteins associated with p‐Tau from PSP samples.

### 2.9 Statistical analysis

For immunoblot analysis, GraphPad Prism (Version 9.4.1, RRID: SCR_002798) was used to determine significance. A one‐tailed *t*‐test was used. Results were considered statistically significant if the *p*‐value was <0.05. Data were not assessed for normality, and no test for outliers was conducted.

For bioinformatics analysis, ingenuity pathway analysis was used to determine significance. Specifically, a right‐tailed Fisher's exact test was used and results were considered statistically significant if the *p*‐value was <0.05. Data were not assessed for normality, and no test for outliers was conducted.

### 2.10 Comparison of BAR‐identified tau proximal proteins to literature and the human protein atlas

Proximal proteins to p‐Tau that were identified in our study were compared to published studies of the Tau interactome in mouse and human brain samples. Published studies using mouse/rat brains were Liu et al., 2016 (Liu et al., 2016), Wang et al., 2017 (Wang et al., 2017), Maziuk et al., 2018 (Maziuk et al., 2018), and Sinsky et al., 2020 (Sinsky et al., 2020), while studies of the Tau interactome in human brains were Meier et al., 2015 (Meier et al., 2015), Ayyadevara et al., 2016 (Ayyadevara et al., 2016), Hsieh et al., 2019 (Hsieh et al., 2019), and Drummond et al., 2020 (E. Drummond et al., 2020). Additional protein interactions were identified by analyzing known protein–protein interactions in IPA or conducting systematic literature searches through PubMed. For systematic literature searches, “tau and a protein of interest were searched together in PubMed.

### 2.11 Enhanced tissue expression and subcellular location

In order to assess if the proteins found associated with p‐Tau had enhanced tissue expression in the brain were compared to normalized Tissue‐Specific RNA expression obtained from V20.1 of the Human protein atlas (RRID:SCR_006710) (Atlas). To understand the normal subcellular distribution of proteins found proximal to p‐Tau in PSP, we compared our significantly enriched list to the subcellular location of these proteins also from V20.1 of the Human protein atlas (Atlas; Thul Peter et al., 2017). Only proteins with an annotation of “approved,” “enhanced,” or “supported” under the “Reliability (IF)” category were included for subcellular location analysis. The cell graphic used in Figure 7b was also retrieved from the Human Protein Atlas.

### 2.12 Enhanced tissue expression and subcellular location

In order to assess if the proteins found associated with p‐Tau had enhanced tissue expression in the brain were compared to normalized tissue‐specific RNA expression obtained from V20.1 of the Human protein atlas (Atlas; Uhlén et al., 2015). To understand the normal subcellular distribution of proteins found proximal to p‐Tau in PSP, we compared our significantly enriched list to the subcellular location of these proteins also from V20.1 of the Human protein atlas (Atlas; Thul Peter et al., 2017). Only proteins with a “Reliability (IF)” annotation of “approved,” “enhanced,” or “supported” were included for subcellular location analysis.

### 2.13 Cell type enrichment

Genes encoding for the proteins identified using BAR were assigned cell‐type specificity based on transcriptomic datasets from the human temporal and motor cortex (Bakken et al., 2020; Zhang et al., 2016). Each gene's mean expression level was standardized across the different cell types, and genes were classified as enriched to a particular cell type with a Z‐score 1.5 and above. Hierarchical clustering analysis with heatmap representation was performed on the standardized datasets in R using “heatmaply” (Galili et al., 2018). Principal component analysis (PCA) was performed on the standardized datasets using “factoextra” (RRID:SCR_016692) and “FactoMineR” (RRID:SCR_014602) libraries in R (Lê et al., 2008).

## 3 RESULTS

### 3.1 BAR selectively biotinylated proteins closely associated with p‐tau in post‐mortem tissue from PSP patients

**FIGURE 1:**
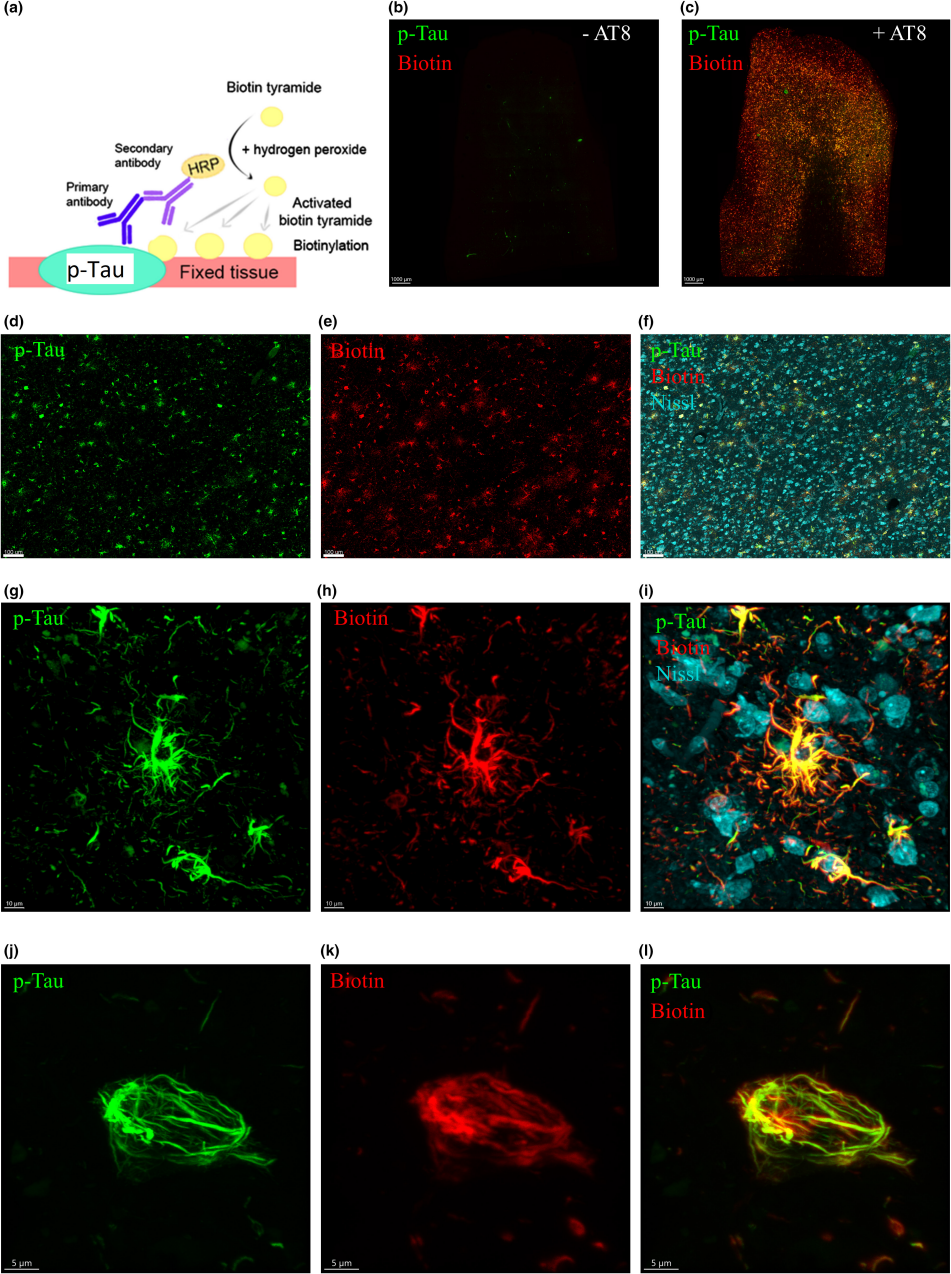
Targeted biotinylation of p‐Tau aggregates in post‐mortem tissue. (a) Process of biotinylation. Primary antibody recognizing p‐Tau was used to target the pathological aggregates of Tau in fixed PSP patient tissue. A secondary antibody conjugated to poly‐HRP was used to facilitate the biotinylation of proteins in proximity to the antibody complex. (b) Biotinylation is negligible when no primary antibody is used to target biotinylation. (c) Biotinylation occurs near Tau aggregates when a primary antibody is applied. Scale bar in (b) and (c) is 1000 μm. (d–f) Biotinylation profile is similar to the phospho‐Tau deposition in the post‐mortem tissue of patients. Scale bar = 100 μm. (g–i) The biotinylation profile is proximal to p‐Tau aggregates in glia with tufted astrocytes and coiled bodies in oligodendrocytes. The scale bar is 10 μm. (j–l) The close proximity of biotinylation in relation to p‐Tau neuronal pathology. Scale bar = 5 μm. This was conducted using tissue available from 4 patients with similar results in each case.

In order to selectively biotinylate proteins in a small radius to phosphorylated Tau in PSP patient post‐mortem tissue, we first targeted phosphorylated forms of Tau using a primary mouse antibody that recognizes p‐Tau at serine 202 (S202) and threonine 205 (T205). p‐Tau pathology has previously been extensively characterized in post‐mortem tissue from PSP patients using the AT8 antibody (Goedert et al., 1995; Malia et al., 2016; Pikkarainen et al., 2010; Williams et al., 2007). Following wash steps, an anti‐mouse secondary antibody conjugated to poly‐HRP was used to bind the primary antibody. Next, biotin‐XX‐tyramide and hydrogen peroxide were added, enabling the biotinylation of proximal proteins by poly‐HRP (Figure 1a). In the negative control group, tissues were processed in the same way except without the addition of the primary antibody. The comparison between the Tau‐targeted samples and negative controls revealed a clear biotinylation pattern. In comparison, there was negligible biotinylated protein detected in the negative controls (Figure 1b,c). Notably, the fluorescent signal of phospho‐Tau as well as biotin was clearly localized within the same structures (Figure 1d–f), demonstrating that our approach to using antibody‐directed biotinylation enables selective and distinct labeling of proteins within a small radius of p‐Tau in glia and neurons (Figure 1g–l).

### 3.2 BAR identifies pathological forms of tau in PSP patients

**FIGURE 2:**
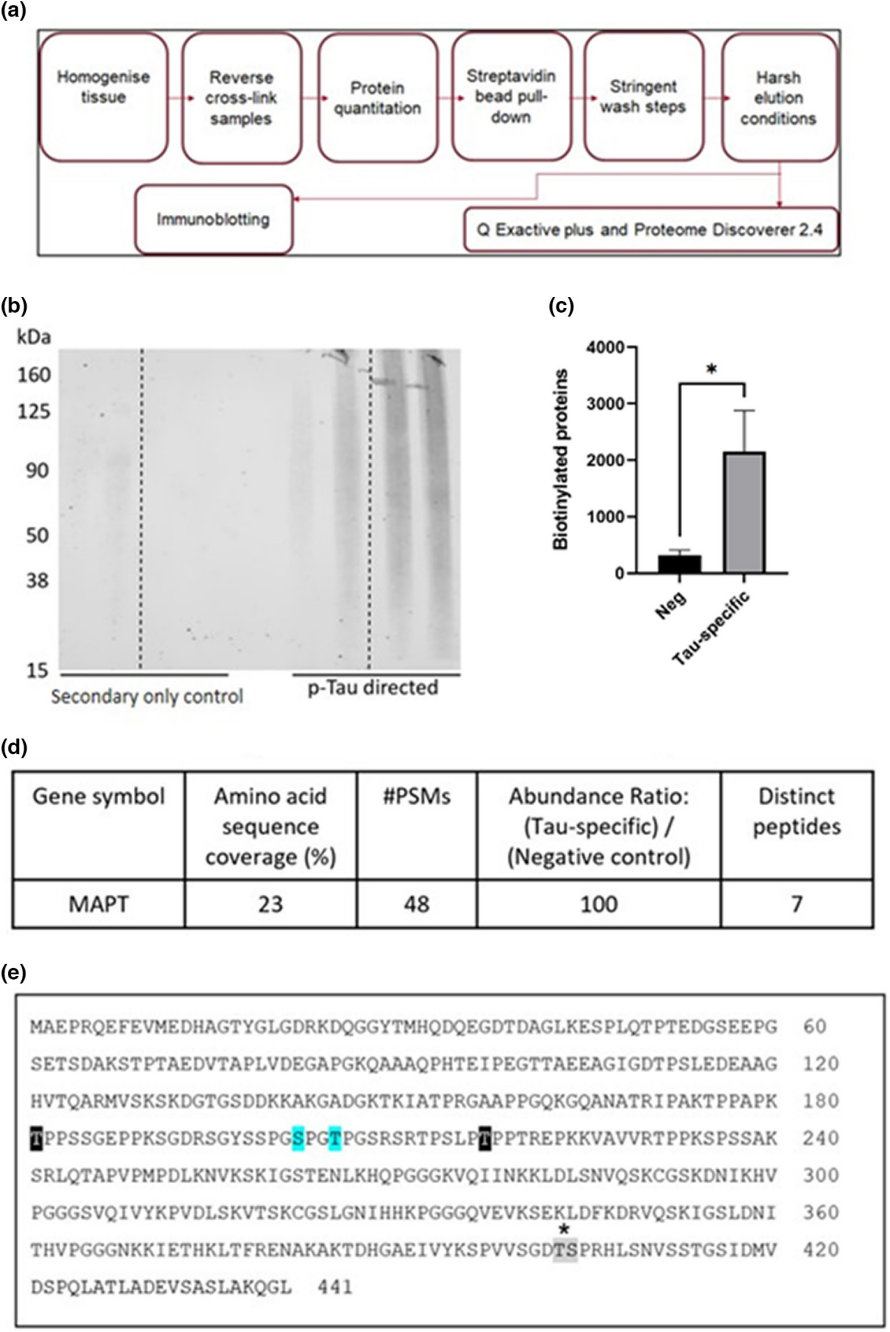
BAR leads to biotinylation of proteins in proximity to p‐Tau. (a) Workflow used to identify Tau interactome in patient tissue. (b) Biotinylation pattern of lysates derived from labeled tissue. *N* = 4 where “*N*” refers to the number of patients. (c) Densitometry of biotinylated profiles in Tau‐targeted samples and negative controls. Student's *t*‐test was conducted on paired samples. Actual *p*‐value = 0.0442, *t*‐value = 2.539, degrees of freedom = 6) (*p < 0.05). *N* = 4 where “*N*” refers to the number of patients. (d) Identification of peptides belonging to Tau. (e) Confidently identified phosphopeptides in PSP patients. Residues highlighted in blue represent the phosphorylation sites recognized by the p‐Tau antibody used. Residues in black boxes refer to phosphorylation sites that were unambiguously assigned. Residues in gray boxes (and with an * above) refer to phosphorylation sites that were assigned to one of two closely positioned amino acids. PSMs – peptide spectrum matches. A 0.01 or 100‐fold abundance respectively indicates the absence or presence of the proximal protein.

In order to identify proximal proteins labeled with biotin, the samples were further processed, and antibody‐targeted Tau was isolated from the complex tissue (Figure 2a). To do this, labeled samples (both control and Tau‐targeted samples) were homogenized and then reverse cross‐linked. The resulting lysates were used to determine whether the biotinylated proteins could be detected by immunoblotting (Figure 2b). Notably, immunoblot analysis of the patient lysates, with streptavidin‐conjugated to a fluorophore, revealed an apparent increase in the levels of biotinylated proteins when compared to the controls (Figure 2c).

To isolate biotinylated proteins, equal amounts of patient samples were incubated with magnetic beads conjugated to streptavidin. The beads were subjected to stringent wash procedures which involved sequential wash steps using high‐salt, high‐pH, and high‐urea buffers to reduce non‐specific binding (Hung et al., 2016). Finally, the isolated proteins were subject to on‐bead trypsin digestion and detected using LC–MS/MS, followed by bioinformatic analyses. To exclude false negatives (non‐specific binding to the beads), we only assessed proteins with at least two unique peptides identified and increased at least 2‐fold compared to the negative control. In total, 117 proteins fit this criterion. To validate our approach in identifying components associated with p‐Tau, we first checked explicitly for amino acid sequences belonging to Tau. As we would expect in tau‐related PSP tissue, we noted an enrichment of Tau peptide sequences compared to the control (Figure 2d). These data clearly indicate the selectivity of this approach.

The MS analysis further allowed us to screen the identified peptides for potential phosphorylation sites. Notably, phosphorylation sites on p‐Tau were found at T403/S404 and T181 (Figure S1) and at T217 (Figure 2e and Figure S1). Notably, all phosphorylation sites differed from the phosphosites recognized by the antibody used (AT8) (Figure 2e).

### 3.3 BAR‐MS identifies known and novel interaction partners of tau

We next compared our putative list of proximal and/or interacting p‐Tau proteins against other studies identifying interaction partners of Tau in mouse [8–10], rat [11], and human samples [12–16], which made use of either immunoprecipitation‐based methods or laser‐capture microdissections along with MS (Table S1). Comparison of our data compared to published datasets or literature searches revealed that approximately 84% of the 117 proteins have previously been found associated with Tau while 16% had not previously been associated with Tau (Table 2, Table S1 novel proteins highlighted). Furthermore, of the proteins associated with p‐Tau in PSP, 15 proteins were found in at least 4 of the 9 MS datasets searched with several protein interactions having been validated using orthogonal methods or found in the insoluble fractions of human neurodegenerative disease tissue or animal models displaying Tauopathies (Table 2). These data demonstrate the ability and application of our BAR‐optimized approach to identifying likely interaction partners of Tau.

**TABLE 2:** Proteins in proximity to p‐Tau using Bar‐MS with at least five additional MS‐based published datasets. “Additional validation” refers to potential interaction partners with Tau that orthogonal methods have validated. “Insoluble in disease states” indicates previously identified proteins in insoluble fractions in neurodegenerative disease states or animal models of Tauopathies. Proteins not found in any MS datasets or previously described as a Tau interactor are italicized. A 0.01 or 100‐fold abundance respectively indicates the absence or presence of the proximal protein.

Gene	Abundance ratio (tau‐specific/control)	MS datasets	Additional validation	Insoluble in disease states
PURA	9.3	(Drummond et al., 2020; Sinsky et al., 2020; Wang et al., 2017)	(McInnes et al., 2018)	
DCTN1	3.7	(Ayyadevara et al., 2016; Hsieh et al., 2019; Sinsky et al., 2020; Wang et al., 2017)	(Haenig et al., 2020; Magnani et al., 2007; Thul Peter et al., 2017)	
TKT	3.7	(Drummond et al., 2020; Sinsky et al., 2020; Wang et al., 2017)		Mouse model of Tauopathy (Pace et al., 2018)
GFAP	3.0	(Ayyadevara et al., 2016; Drummond et al., 2020; Hsieh et al., 2019; Maziuk et al., 2018; Sinsky et al., 2020)		
SYNJ1	2.8	(Drummond et al., 2020; Hsieh et al., 2019; Meier et al., 2015; Sinsky et al., 2020; Wang et al., 2017)	(Ando et al., 2020; McInnes et al., 2018)	AD brains (Ando et al., 2020; Kepchia et al., 2020; McInnes et al., 2018) and mouse model of Tauopathy (Pace et al., 2018)
YWHAG	2.6	(Ayyadevara et al., 2016; Drummond et al., 2020; Hsieh et al., 2019; Sinsky et al., 2020; Wang et al., 2017)		
MAP1A	2.6	(Ayyadevara et al., 2016; Drummond et al., 2020; Sinsky et al., 2020)	(Alonso et al., 1997)	Asymptomatic AD (Hales et al., 2016)
ENO2	2.5	(Drummond et al., 2020; Hsieh et al., 2019; Liu et al., 2016; Pires et al., 2019; Sinsky et al., 2020)		
PFKM	2.5	(Drummond et al., 2020; Hsieh et al., 2019; Maziuk et al., 2018; Sinsky et al., 2020; Wang et al., 2017)		
NSF	2.3	(Drummond et al., 2020; Hsieh et al., 2019; Sinsky et al., 2020)		
UBA1	2.1	(Ayyadevara et al., 2016; Drummond et al., 2020; Hsieh et al., 2019; Sinsky et al., 2020; Wang et al., 2017)		AD brains (Kepchia et al., 2020) and mouse model of Tauopathy (Pace et al., 2018)
YWHAZ	2.1	(Drummond et al., 2020; Pace et al., 2018; Sinsky et al., 2020; Wang et al., 2017)	(Hashiguchi et al., 2000; Jaesun et al., 2004; Li & Paudel, 2016; Matthews & Johnson, 2005; Nellist et al., 2002; Papanikolopoulou et al., 2018; Qureshi et al., 2013; Sadik et al., 2009; Sluchanko et al., 2009; Tugaeva et al., 2017; Yuan et al., 2004)	
SPTAN1	2.1	(Drummond et al., 2020; Hsieh et al., 2019; Sinsky et al., 2020; Wang et al., 2017)		
VAMP2	2.0	(Drummond et al., 2020; Hsieh et al., 2019; Sinsky et al., 2020)		
PGK1	2.0	(Drummond et al., 2020; Hsieh et al., 2019; Sinsky et al., 2020; Wang et al., 2017)		
Not previously associated with Tau				
*ACTG2*	100			
*CPT1A*	100			
*ECH1*	100			
*HSD17B4*	100			
*MYH1*	100			
*RAB6A*	100			
*TUBAL3*	100			
*GSTO1*	11.9			
*PMP2*	6.6			AD (Hales et al., 2016)
*SEPT3*	3.7			
*MAOA*	3.6			Mild cognitive impairment (Hales et al., 2016)
*DDAH2*	3.2			
*FTL*	3.1			PSP and CBD (Ebrahim et al., 2011)
*ARF5*	3.0			
*NDUFB7*	2.6			
*MOG*	2.3			Mild cognitive impairment (Hales et al., 2016)
*MAOB*	2.3			
*COX6B1*	2.2			
*ME3*	2.0			

### 3.4 Bioinformatic analysis of proteins in proximity to tau is enriched for cellular assembly and organization

**FIGURE 3:**
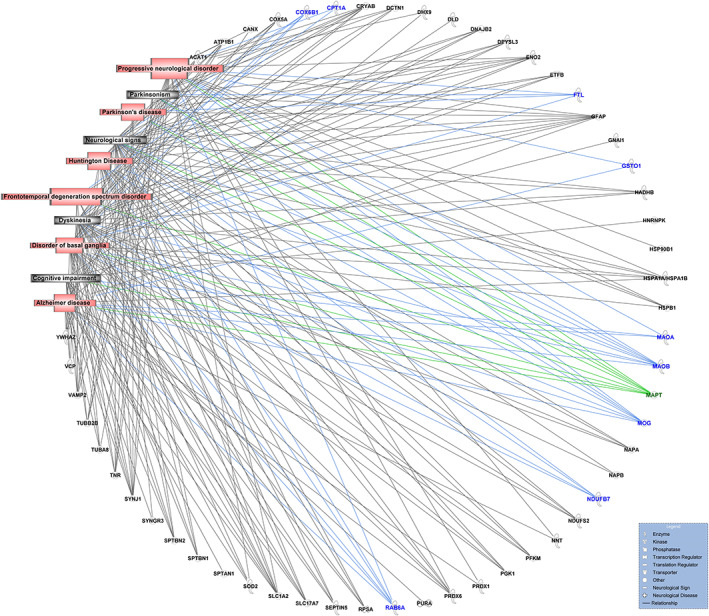
Proteins in proximity to Tau are enriched for known Tau interactions and proteins linked to neurological dysfunction. Groups of at least 10 proteins found in proximity to p‐Tau (ratio p‐Tau/control ≥2‐fold) that are known to be associated with neurological disorders – Frontotemporal degeneration spectrum disorder, Parkinson's disease, parkinsonism, Alzheimer disease, Huntington Disease, and disorder of the basal ganglia in red or neurological signs associated with PSP such as dyskinesia and cognitive impairment highlighted in gray. How Tau is related to these neurological dysfunctions and disorders is highlighted in green, while previously unreported proteins identified in close proximity to p‐Tau from Figure 4a are highlighted in blue. Results were obtained from proteomic studies using tissue from 4 different patients.

In order to gain further insight into the functionality of proteins in proximity to p‐Tau, we analyzed the 117 BAR‐MS identified proteins (stringent criteria of ≥2 fold‐change compared to the control and 2 or more unique peptides) using IPA. Notably, the most significant “Diseases and Disorders” function associated with the 117 proteins was “Neurological Disease” (*p*‐value range: 3.05E‐03–4.17E −23, Number of Molecules: 87, 74% of total identified). Further analysis of this pathway identified 37 groups of at least 10 proteins that were significantly associated with “Neurological Disease” including groups of proteins associated with other neurological disorders such as AD (1.28E‐08), disorder of basal ganglia (4.2E‐19), progressive neurological disorder (1.43E‐09), Huntington disease (7.73E‐15), Parkinsonism (3.18E‐7), Parkinson's disease (1.39E‐6), and frontotemporal degeneration spectrum disorder (4.5E‐4). While neurological signs associated with PSP, such as dyskinesia (1.01E‐15) and cognitive impairment (2.06E‐3) (Figure 3), also indicated enrichment of proteins known to associate with Tau and with involvement in neurological dysfunction.

**FIGURE 4:**
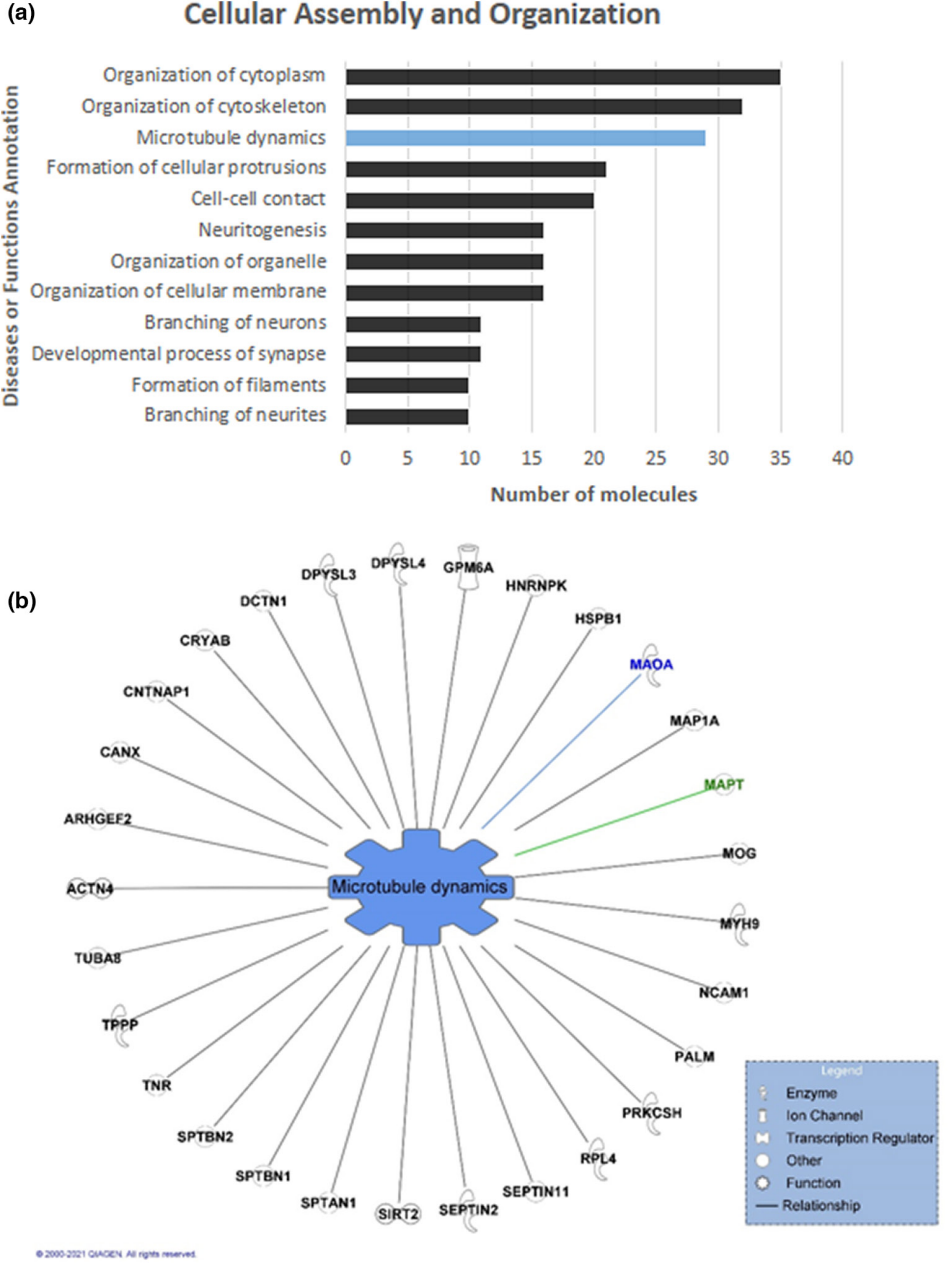
Grouping of proteins associated with Cellular Assembly and Organization (a) Groups of at least 10 proteins found in proximity to p‐Tau (ratio p‐Tau/control ≥2‐fold) associated with Cellular Assembly and Organization subfunctions, highlighted in blue is further broken down in (b). (b) Individual proteins in proximity to p‐Tau (ratio p‐Tau/control ≥2‐fold) were identified in a network associated with Microtubule dynamics. MAPT is highlighted in green, while a previously unreported protein identified proximal to p‐Tau from Figure 2a is highlighted in blue. Results were obtained from proteomic studies using tissue from 4 different patients.

Notably, the most statistically significant molecular and cellular functions identified by IPA were “Cellular Assembly and Organization” (59 Molecules, *p*‐value range = 3.02 E‐03 – 3.54 E‐11). Accordingly, we further analyzed the proteins associated with this function revealing multiple groups of proteins (here we show functions containing at least 10 proteins) associated with “Cellular Assembly and Organization” (Figure 4a). Furthermore, further analysis of the subfunction named “Microtubule dynamics” revealed Tau alongside 28 additional proteins with roles associated with microtubule dynamics (Figure 4b). Here, the identification of clusters of proteins associated with microtubule dynamics was expected, given the role of Tau as a microtubule‐associated protein. Thus, again indicating that our proximity‐labeling method was reliable for identifying p‐Tau alongside major complexes (and associated proteins) known to interact with Tau.

**FIGURE 5:**
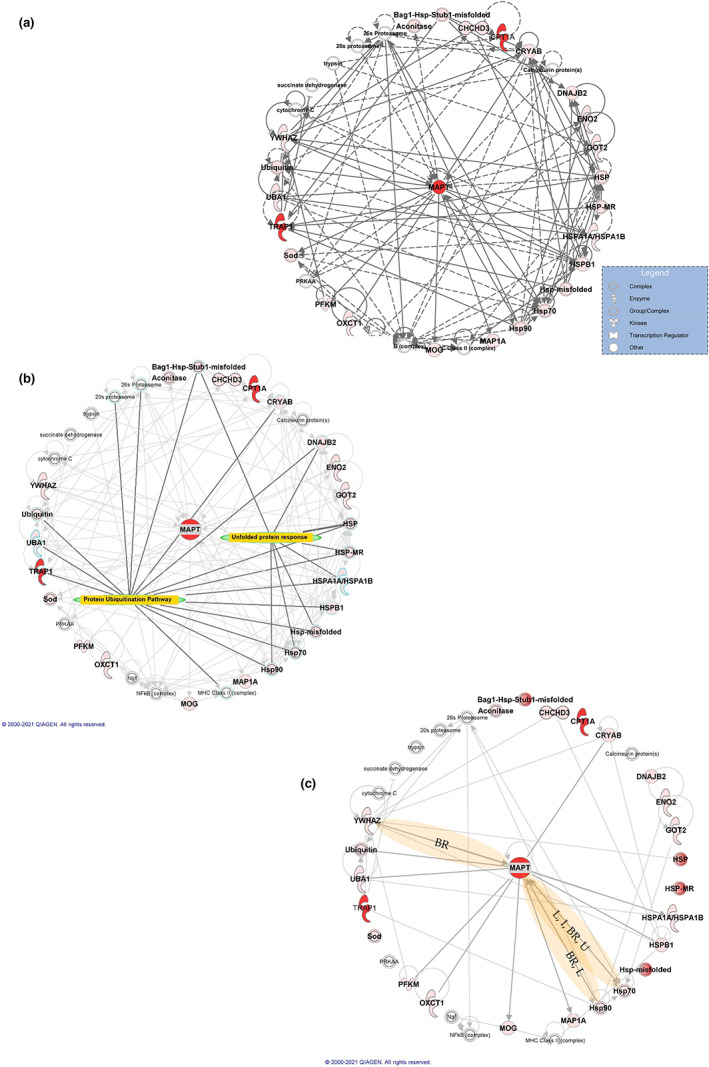
Protein networks associated with Tau. (a) Overlay of canonical pathways, “Protein Ubiquitination Pathway” and “Unfolded protein response.” (b) Proteins in proximity to p‐Tau in PSP brain tissue associated with the network: “Hereditary Disorder, Organismal Injury and Abnormalities, Skeletal and Muscular Disorders.” Increasing shades of red indicate a higher fold‐change ratio compared to the control. Double circles represent protein complexes. (c) Known protein–protein interactions within the network. Darker lines represent protein–protein interactions with Tau. Yellow highlights represent proteins with other relationships with Tau. BR, binding regulator; L, localization; U, ubiquitination, and I, inhibition. Results were obtained from proteomic studies using tissue from 4 different patients.

To gain insight into the protein network relationships in proximity to p‐Tau, IPA was used to identify networks of proteins with known relationships. Notably, Tau was found in a network associated with “Hereditary Disorder, Organismal Injury and Abnormalities, Skeletal and Muscular Disorders” (Figure 5a). To determine whether this network was enriched for any particular biological functions, IPA was used to overlay Canonical Pathways (Figure 5b). The pathway with the most significant number of molecules was the protein ubiquitination pathway, with 16 of the 30 proteins associated with this pathway. In addition, another pathway identified was the Unfolded protein response (UPR) pathway (7 proteins). Together, the identification of these pathways revealed associated networks of proteins in proximity to p‐Tau related with protein degradation systems and protein folding functions implicated in PSP and other neurodegenerative conditions.

Next, the protein network was used to analyze known protein–protein interactions (Figure 5c). This analysis revealed 12 proteins/protein complexes interacting with Tau (CRYAB, HSPA1A/HSPA1B, HSPB1, MAP1A, MOG, OXCT1, PFKM, TRAP1, UBA1, YWHAZ, as well as complexes, HSP70, HSP90, Hsp‐misfolded, and Ubiquitin). Of the known interaction partners, further analysis of each protein–protein interaction using IPA revealed a subset of proteins/protein complexes that have other known relationships with Tau (Figure 5c). These included components of the HSP70 complex, which could be a binding regulator of Tau, inhibit Tau function, and influence the localization and ubiquitination of Tau. In addition, proteins that form part of the HSP90 complex were also identified, influencing the localization of Tau and acting as a Tau binding regulator. Another known interaction partner of Tau in the network was YWHAZ, which is known to act as a binding regulator of Tau.

### 3.5 Proximal proteins to p‐Tau in PSP display enhanced brain expression and multiple subcellular localizations

**FIGURE 6:**
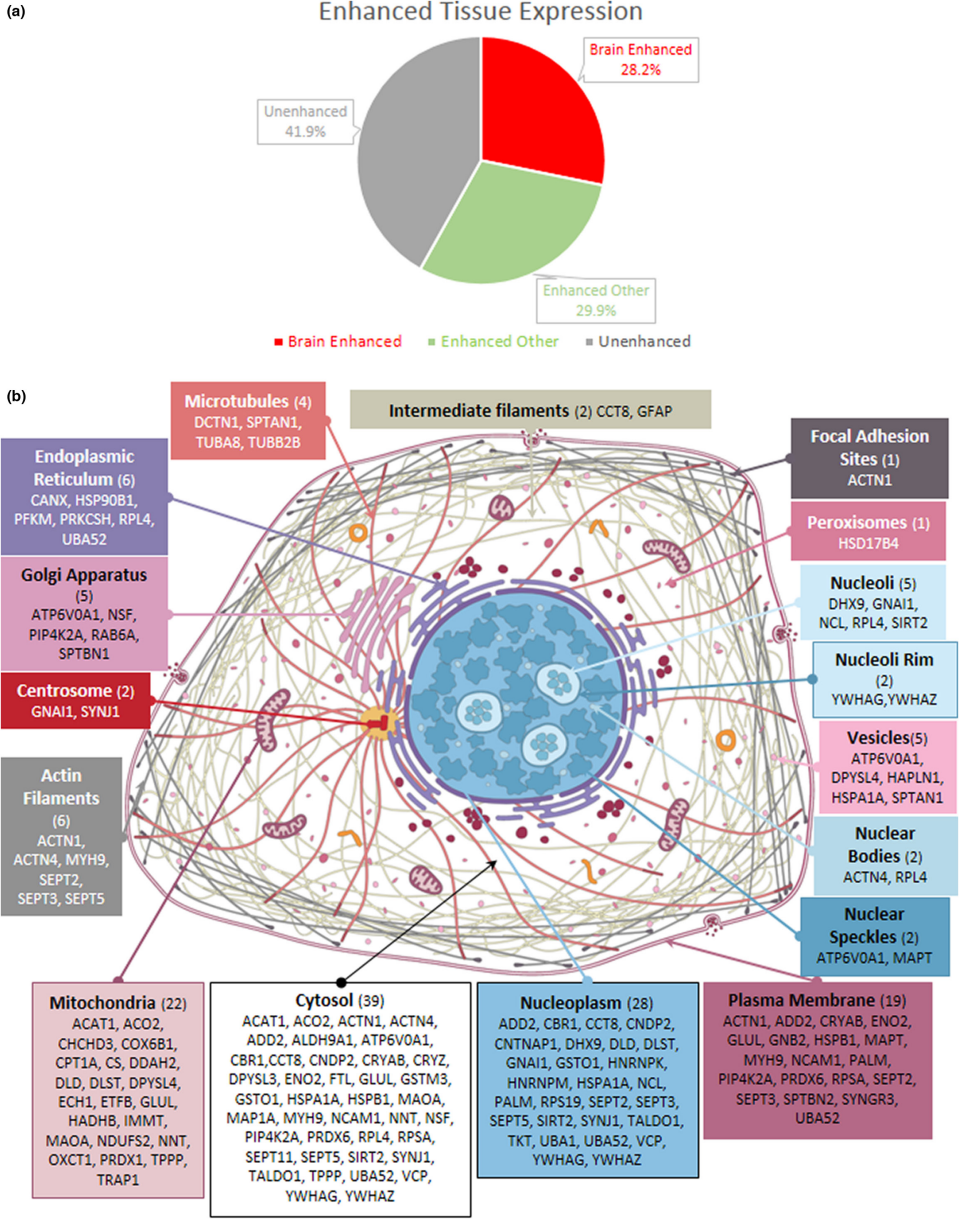
Brain enhanced expression and subcellular location of proximal proteins associated with p‐Tau in PSP. (a) of the 117 proteins associated with p‐Tau in PSP, 28.2% exhibited enhanced brain RNA expression shown in red. Proteins with tissue enhanced RNA expression in tissues other than the brain is green, while proteins with no enhanced RNA expression are shown in gray. (b) depicts subcellular locations of proteins associated with p‐Tau in PSP. The number of proteins found in each subcellular location is indicated with (=N). Results were obtained from proteomic studies using tissue from 4 different patients.

Next, we sought to identify if proteins found proximal to p‐Tau in PSP showed enhanced expression in the brain compared to other tissue types. We used a bioinformatic approach to extract RNA‐seq data generated in the Human Protein Atlas (Atlas; Uhlén et al., 2015). We found that 28.2% of the 117 significantly p‐Tau‐associated proteins display enhanced brain expression relative to multiple tissues sampled, indicating that these proteins may have an essential role in brain function (Figure 6a). Additionally, we utilized the Human Protein Atlas cell immunofluorescence data to observe the subcellular location of the proteins associated with p‐Tau in PSP (Figure 6b). Eighty‐six proteins had sufficient data to be allocated subcellular locations, and Figure 6b shows that proteins associated with p‐Tau are from multiple subcellular compartments despite p‐Tau pathology in PSP residing overwhelmingly in the cytoplasm (Kovacs, 2015).

### 3.6 Enriched cell‐type expression of proteins proximal to p‐Tau in PSP

**FIGURE 7:**
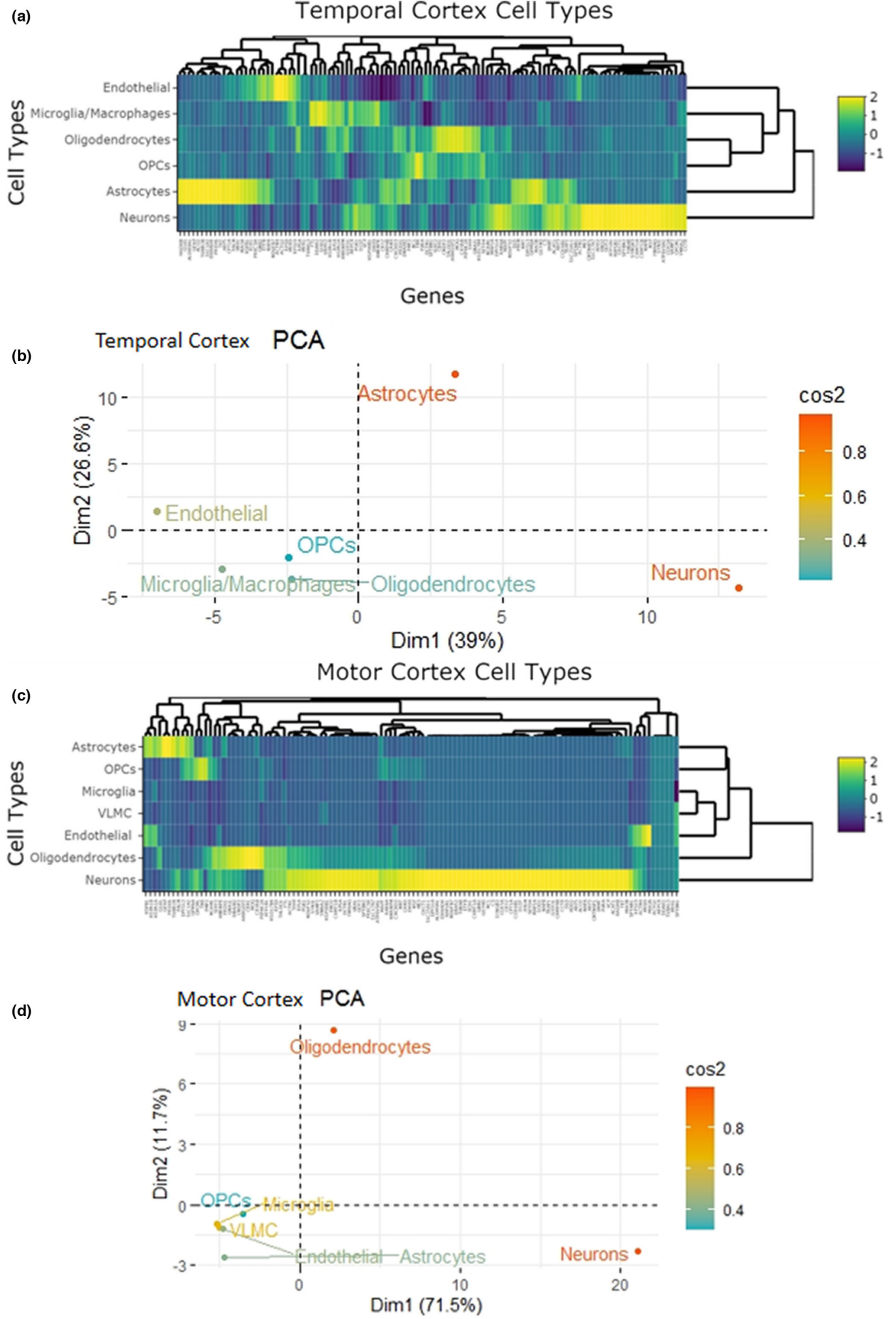
Predicted cell‐type enrichment of proteins found in proximity to p‐Tau (ratio p‐Tau/control ≥2‐fold) based on standardized human RNA‐seq datasets. (a) and (b) were constructed using an RNA‐seq dataset with cells sampled from the human temporal cortex (Zhang et al., 2016). (a) hierarchical cluster analysis and heatmap representation for transcriptional expression of proteins found proximal to p‐Tau in neurons, astrocytes, oligodendrocytes, oligodendrocyte precursor cells (OPCs), microglia, and endothelial cells. Z‐scores above 1.5 with a predicted enrichment are shown in green‐yellow. (b) principal component analysis (PCA) shows that the predicted expression variance of neurons and astrocytes is primarily explained in the first and second principal components. (c) and (d) were constructed using an RNA‐seq dataset with cells sampled from the human motor cortex (Bakken, Jorstad et al. 2020). (c) hierarchical cluster analysis and heatmap representation for transcriptional expression of proteins found proximal to p‐Tau in neurons, astrocytes, oligodendrocytes, oligodendrocyte precursor cells (OPCs), microglia, endothelial and vascular and leptomeningeal cells (VLMC). Z‐scores above 1.5 with a predicted enrichment are shown in green‐yellow. (d) principal component analysis (PCA) shows the predicted expression variance in the first and second dimensions are primarily explained by neurons and oligodendrocytes indicated by cos2. Results were obtained from proteomic studies using tissue from 4 different patients.

Next, to determine if any of the proteins identified using BAR could be associated with specific cell types, we compared our list of significantly associated p‐Tau proteins with two RNA expression datasets from the human temporal and motor cortices (Bakken et al., 2020; Zhang et al., 2016). Comparing our list of p‐Tau‐associated proteins to the temporal cortex dataset, we found 75 of the 117 proteins predicted to be enriched in various cell types. Thirty‐four genes in neurons, 22 in astrocytes, 8 in oligodendrocytes, 5 in microglia, 4 in endothelial cells, and 2 in oligodendrocyte precursor cells (OPCs) (Table S1) displayed a predicted enrichment represented by the hierarchical clustering heatmap in Figure 7a. The variance was primarily explained by genes significantly enriched in neurons and astrocytes, shown via the PCA biplot of cell types in Figure 7b. Comparatively, we found 101 of the significantly associated p‐Tau proteins to be in five cell types in the motor cortex transcriptomic dataset. Seventy‐six genes were enriched in neurons, 11 in oligodendrocytes, 9 in astrocytes, 3 OPCs, and 2 in endothelial cells (Table S1) and visually displayed by Figure 7c. Figure 7d shows that the variance is described mainly by genes enriched primarily in neurons and, to a lesser extent, oligodendrocytes. By comparing the two RNA‐seq datasets, 68 genes were enriched in both datasets (Figure S2) while 47 genes were enriched in the same cell type in both datasets, 32 in neurons (Figure S2), 7 in astrocytes (Figure S2), 6 in oligodendrocytes (Figure S2), and one for OPCs and endothelial cells, respectively (Table S1). Discrepancies between the RNA‐seq datasets can be explained by the different methods employed (e.g., immunopanning for temporal cortex vs. single nuclei RNA‐seq for motor cortex) and differences in expression in anatomical brain regions (Lake Blue et al., 2016; Saliba et al., 2014). This cell enrichment reflects the cell‐type Tau pathology seen in PSP, which occurs in neurons, astrocytes, and mature oligodendrocytes (Ahmed et al., 2013; Gabor G. Kovacs et al., 2020).

### 3.7 Bioinformatic analysis shows pathways enriched for protein degradation, stress responses, cytoskeletal dynamics, metabolism, and neurotransmission

**FIGURE 8:**
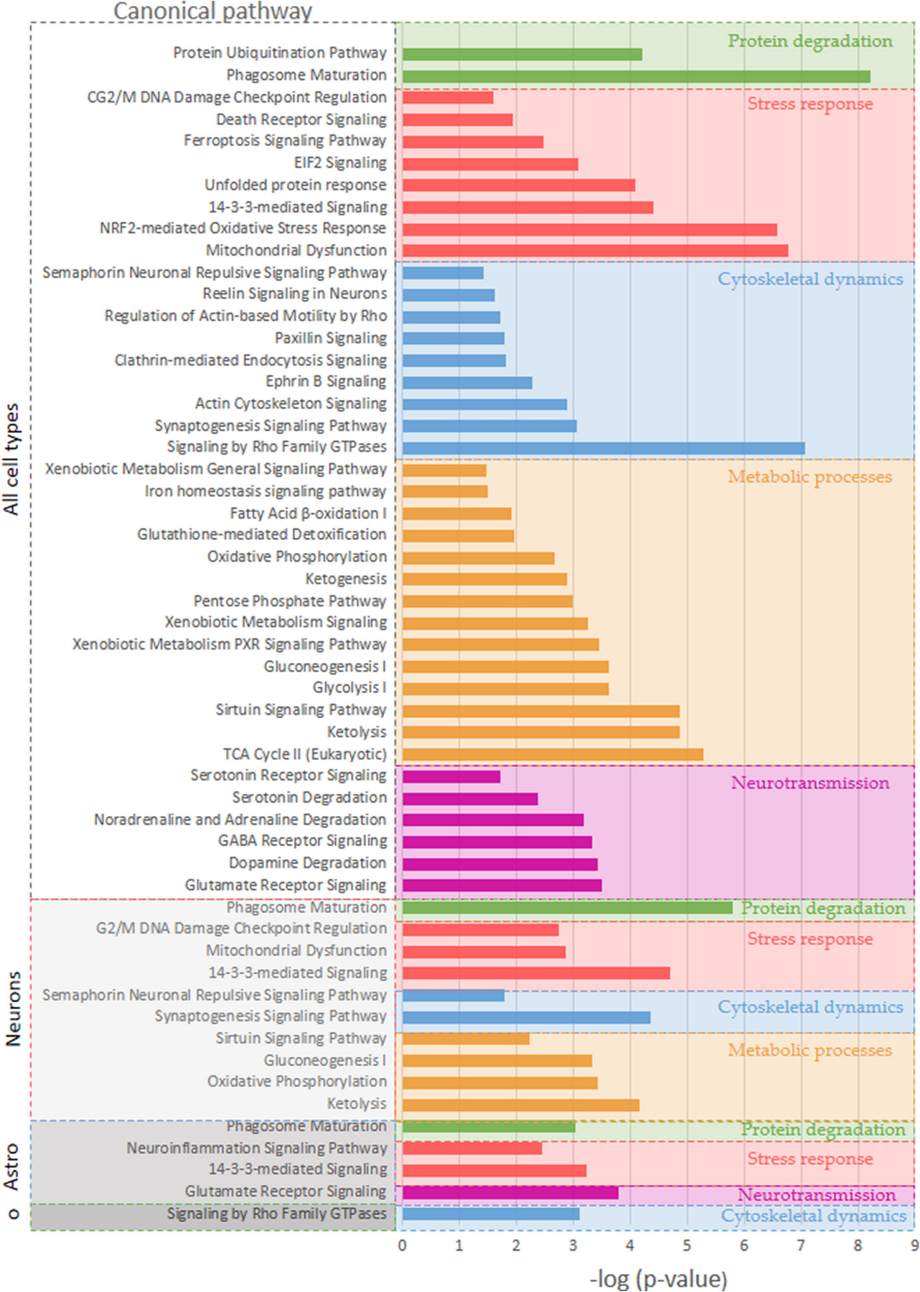
Selection of statistically significant canonical pathways associated with proteins found in proximity to p‐Tau (ratio p‐Tau/control ≥2‐fold). Pathways highlighted in green represent pathways associated with protein degradation systems, while those highlighted in red, blue, orange, and purple are associated with stress responses, cytoskeletal dynamics, metabolic processes, and neurotransmission. Particular pathways predicted to be enriched in neurons, astrocytes (Astro), and oligodendrocytes (o) are also displayed. Results were obtained from proteomic studies using tissue from 4 different patients.

Finally, to determine additional canonical pathways that could be affected by proteins in proximity to p‐Tau, IPA was used to identify statistically enriched molecular canonical pathways. Notably, subsets of canonical pathways with related functions could be identified. These identified canonical pathways involved in protein degradation systems (including the proteasome and autophagy), stress responses (UPR and EIF2 signaling), cytoskeletal dynamics (including proteins which are involved in Actin‐based motility and signaling, synaptogenesis and endocytosis signaling pathways), metabolic processes, and neurotransmission. These data indicate potential perturbations in protein degradation systems, stress responses, cytoskeletal dynamics, metabolism, and neurotransmission associated with p‐Tau aggregation (Figure 8).

Together, IPA analysis of enriched “Molecular and Cellular Functions” along with “Canonical Pathways” and interaction networks of Tau revealed clusters of proteins that were associated with cellular organization systems, protein clearance, stress response pathways, metabolic processes, and neurotransmission providing further insight into the individual proteins and protein complexes in proximity to pathological forms of Tau from the post‐mortem tissue of PSP patients. Additionally, cell enrichment showed that some of these pathways are predicted to be enriched in neurons, astrocytes, and oligodendrocytes based on RNA‐seq data.

## 4 DISCUSSION

As a proof‐of‐concept study to determine proximal or interacting proteins to p‐Tau in tissue pathology, we applied the BAR method and MS for unbiased, in situ identification of pathological p‐Tau aggregates and co‐aggregating protein partners in a small subset of PSP patient tissue. We identified 117 proteins of which >80% of the proteins have previously been associated with Tau while an extensive network of proteins has previously been associated with Tauopathies and known complexes associated with Tau. Furthermore, higher confidence p‐Tau proximal proteins included previously associated with Tau in multiple publications. These included 15 proteins that were found associated with Tau in at least four other MS‐based publications. Notably, in addition to being identified in four MS datasets, the interaction between Tau and YWHAZ (14‐3‐3ζ) was also found by additional publications (Hashiguchi et al., 2000; Jaesun et al., 2004; Li & Paudel, 2016; Matthews & Johnson, 2005; Nellist et al., 2002; Papanikolopoulou et al., 2018; Qureshi et al., 2013; Sadik et al., 2009; Sluchanko et al., 2009; Tugaeva et al., 2017; Yuan et al., 2004). This demonstrates the evident selectivity of this method to enrich interaction partners of a selected protein of interest, in this instance, p‐Tau from PSP cases.

In addition to identifying known interaction partners of Tau, we were also able to identify proteins that have additional relationships with Tau, some of which directly relate to features of Tau pathology, including aggregation, ubiquitination, and Tau association with microtubules. Proteins that form part of HSP90/70 complexes and YWHAG (14‐3‐3γ) were found to have additional relationships with Tau. Dou et al. (2003) demonstrated that reduced expression of HSP70 and HSP90 using siRNA leads to the accumulation of aggregated, phosphorylated Tau and reduced binding of Tau with microtubules. HSP70 proteins have been reported to increase inhibition of phosphorylated active Tau and have also been associated with Tau ubiquitination as HSP70 proteins and CHIP can increase ubiquitination of Tau (Carrettiero et al., 2009; Muchowski & Wacker, 2005). YWHAZ has been reported to bind and mediate the phosphorylation of Tau (Yuan et al., 2004). Together, the identification of known interaction partners of Tau and modifiers of Tau pathology demonstrates the ability of this method to characterize the web of protein interactions associated with p‐Tau, including proteins that may have a role in altering the pathological features of Tau.

Additionally, with datasets obtained from the Human Protein Atlas, we assessed that 28.21% of proteins associated with p‐Tau in PSP displayed enhanced RNA expression in the brain and typically are associated with various subcellular locations despite p‐Tau pathology residing overwhelmingly in the cytoplasm (Atlas; Kovacs, 2015; Orr et al., 2017; Thul Peter et al., 2017; Uhlén et al., 2015). The presence of proteins typically associated in subcellular locations other than the cytosol indicates recruitment or sequestration of some of these proteins warrants further investigation as modulating this phenomenon may influence the development of pathology and dysfunction in PSP. Using a similar approach, we sought to identify if proteins associated with p‐Tau were enriched in particular cell types by comparing them to two RNA‐seq datasets from the human temporal and motor cortices (Bakken et al., 2020; Y. Zhang et al., 2016). We identified that the transcriptomic profile indicates that these proteins are enriched particularly in neurons, astrocytes, and mature oligodendrocytes, reflecting the cell type Tau pathology of NFTs, TAs, and CBs seen in PSP (Ahmed et al., 2013; Kovacs et al., 2020).

An excellent example of this is FTL, where it is predicted to be enriched in microglia from the temporal cortex dataset, which is in line with IHC studies in humans showing it as a marker of microglia; however, it has been shown to be up‐regulated in astrocytes in PSP patients with a subset of TAs displaying colocalization, which is also an independent validation of our finding (Ebrahim et al., 2011; Kaneko et al., 1989). Interestingly, TAs have been positively associated with the expression of the microglial gene‐enriched immune network, so further investigation if astrocytes are directly influencing this by expressing these genes or having a complex interaction with microglia to have an impact on TA pathology and PSP pathogenesis (Allen et al., 2018).

We gained further insight using IPA into the canonical pathways associated with proteins in proximity to p‐Tau. Using this workflow, we consistently identified groups of proteins affecting protein degradation systems, stress responses, cytoskeletal dynamics, metabolic processes, and neurotransmission. Notably, the protein degradation system and unfolded protein response pathways were also identified in the network of protein–protein interactions that connected back to Tau, demonstrating the ability to identify networks of protein–protein interactions that link to Tau and the downstream processes associated with these networks. The ability to identify and characterize networks of proteins associated with p‐Tau has essential implications for understanding neurodegenerative processes as it may help identify changes to molecular pathways that occur early during pathogenesis. Furthermore, identifying proteins and pathways is vital as the complete cellular mechanisms involved in p‐Tau‐induced onset, cellular dysfunction, and cell death are not fully understood (Orr et al., 2017).

IPA of proteins associated with p‐Tau in PSP found canonical pathways associated with protein degradation, stress responses, metabolism, and neurotransmission. Two stress response pathways, UPR and EIF2 signaling have been associated with PSP via GWAS. A key regulator of UPR and EIF2 signaling, *EIF2AK3*, was identified as a GWAS risk factor giving rise to a hypomorphic allele, leading to increased neuronal vulnerability to endoplasmic reticulum stress and Tau accumulation (Höglinger et al., 2011; Stutzbach et al., 2013; Yuan et al., 2018). One of the components of the UPR, also involved in phagosome maturation which we found to associate with p‐Tau in PSP, is VCP. VCP has been shown to regulate Tau accumulation via its disaggregase activity, and a missense mutation (D395G) leading to decreased disaggregase activity has been identified in patients with frontotemporal degeneration displaying prominent Tau pathology and vacuolation. Thus, indicating modulating the recruitment of VCP to p‐Tau pathology in PSP may be a way of decreasing Tau pathology (Darwich et al., 2020). Another protein we found associated with p‐Tau in PSP involved in the phagosome maturation pathway is NSF. NSF has been linked with PSP and Tau pathology because of an *NSF* exonic polymorphism within the *MAPT* H1 haplotype being associated with PSP, CBD, and FTD risk (Pastor et al., 2004; Yokoyama et al., 2017). This exonic polymorphism in *NSF* has been associated with a more significant Tau pathology in PSP, particularly Tau threads (Allen et al., 2018). NSF is required for vesicle‐mediated transport required for phagosome maturation, synaptic vesicle release, and neurotransmission, indicating it may be involved in multiple pathways implicated in PSP (Rizo & Xu, 2015).

Furthermore, synaptic vesicle release and neurotransmission are implicated by SYNJ1 being associated with p‐Tau. SYNJ1 is involved in clathrin‐mediated endocytosis and is essential for synaptic vesicle dynamics. Autosomal recessive mutations lead to early‐onset atypical parkinsonism with uncharacterized neuropathology or epileptic encephalopathy with neuronal tau pathology indicating loss of SYNJ1 function is associated with Tau neuronal pathology and development of atypical parkinsonism (Hardies et al., 2016; Schneider & Alcalay, 2017). Thus, the sequestration of SYNJ1 in p‐Tau pathology could potentially influence dysfunction and exacerbate neuronal tau pathology formation in PSP. Additionally, genes associated with synaptic processes have been shown to be positively associated with tau neurofibrillary tangle (NFT) pathology in PSP, indicating altered synaptic function is linked to neuronal Tau pathology in PSP and warrants further investigation (Allen et al., 2018).

Additionally, the influence of alterations to neurotransmission and metabolic processes is highlighted by our finding of MAOB associated with p‐Tau pathology, which previously has not been associated with p‐Tau interaction via MS. MAOB is a primary amine oxidase that is important for the catabolism of dopamine and is primarily expressed by astrocytes and a subpopulation of neurons in the central nervous system (Ekblom et al., 1993). Quantitative western blot analysis showed MAOB is increased in PSP in regions associated with degeneration (Tong et al., 2015, 2017). A randomized, placebo‐controlled Phase‐III clinical trial into a MAOB inhibitor rasagiline was conducted in a PSP cohort (Nuebling et al., 2016). While the trial was underpowered and failed to see a significant influence on the primary endpoint, post hoc analysis indicated a beneficial effect on limb motor function, which may implicate MAOB on influencing specific symptoms seen in PSP.

Some advantages of the BAR method over traditional fractionation methods and immunoprecipitations are the ability to specifically label proteins (Killinger et al., 2022) within a small labeling radius to the protein‐of‐interest (such as p‐Tau), followed by homogenization that uses comparatively less sample amount. Using vibratome‐prepared tissue also enhances antigenicity and reduces the chance of molecular extraction compared with paraffin‐embedded samples and a validated p‐Tau antibody resistant to over fixation, which can occur in human post‐mortem samples (Pikkarainen et al., 2010; Ramos‐Vara, 2005). However, the technique is amendable to use paraffin‐embedded samples (Bar et al., 2018). This unique characteristic significantly increases the chances of identifying proteins entangled or within proximity of the pathological Tau, which is not possible with other isolation and proteomic approaches such as laser microdissection. In addition, the covalent attachment of biotin to these proteins means that the proteins can later be isolated in more harsh buffers enabling extraction of proteins that would not be soluble using standard immunoprecipitation buffers. The ability to identify protein‐interaction partners from fixed post‐mortem tissue also means that these can be evaluated using tissue precisely from multiple central nervous system regions (allowing further interrogation of the unique aggresomes occurring throughout the central nervous system). Therefore, this methodology is highly applicable to other neurodegenerative diseases characterized by the aggregation of proteins such as α‐synuclein and TDP‐43, which have well‐characterized antibodies available.

A limitation of the BAR technique is that labeling resolution does not allow direct interactors of p‐Tau to those of proximal proteins, so independent methods such as IP may be required for confirmation (Bar et al., 2018). However, the labeling radius can be reduced by using smaller identifying antibodies such as nanobodies or antibodies directly conjugated with HRP. We sought to overcome this with the bioinformatic analysis outlined above to gain greater insight into potential direct interactors with p‐Tau in PSP. Given the availability of motor cortex samples at the time of the study, it should be acknowledged that increasing number of cases may provide deeper proteome coverage of proximal p‐Tau proteins. However, the purpose of this study was to demonstrate the utility of BAR for identifying aggresomal protein signatures that may (i) help to identify other aggregation‐prone proteins in pathology, (ii) distinguish unique signatures that differentiate between clinical subtypes, and (iii) give insights into the cellular mechanisms that are perturbed during the onset of disease.

The importance of analyzing the protein aggregates for each neurodegenerative disease can be highlighted by a recent study by Pires and colleagues who demonstrated that Secernin 1 co‐localizes with p‐Tau in cases of AD but not other Tauopathies including PiD, CBD, or PSP (Pires et al., 2019). While another study by Prikas et al (Prikas et al., 2022) identified that tau interacts with N‐ethylmaleimide sensitive fusion protein (NSF) ATPase to regulate AMPA receptor trafficking in a dose‐dependent manner in mutant tau and Alzheimer's mouse models. Taken together, these studies suggest that the composition of co‐aggregating proteins between Tauopathies may diverge, and there may be other unidentified proteins that may differentiate one Tauopathy from another. Additionally, biochemical and ultrastructural analysis of Tauopathies shows that the composition of Tau, which makes up the neuropathology, is distinctly different and specific for each Tauopathy (Falcon et al., 2019; Falcon, Zhang, Murzin, et al., 2018; Falcon, Zhang, Schweighauser, et al., 2018; Shi et al., 2021; Zhang et al., 2020). Thus, identifying novel aggregate components in human tissue is an important step that will help understand molecular pathways altered in specific Tauopathies. Together, identifying the common and unique aggregate components may help to reveal further insight into the converging and diverging mechanisms that underpin Tauopathies and other closely related neurodegenerative diseases. Future studies comparing different Tauopathies in multiple brain regions would help elucidate these conserved and unique components and subsequent pathways, revealing molecular drivers of pathology and potential therapeutic approaches targeting these components.

Overall, this approach will serve as a complementary method to classical fractionation and immunoprecipitation, IHC, and proximity‐ligation methods and may assist in identifying aggregating proteins beyond Tau, which is particularly pertinent in numerous neurodegenerative conditions. This, in turn, will assist in identifying other proteins (and molecular functions) involved in PSP and other forms of neurodegeneration.

## 5 CONCLUSIONS

Finding and validating aggregate components has thus far been a slow process, and in many instances, their identification has been fortuitous. The identification of these components, however, is integral to understanding the molecular underpinnings of disease. Here, we demonstrate an unbiased approach for accurately labeling proteins within a small radius of p‐Tau directly from PSP patient post‐mortem tissue facilitating multiple proximal proteins to be quickly identified by MS. Bioinformatic analysis showed the multi‐cellular contribution to PSP pathway and highlighted canonical pathways including protein degradation, stress responses, cytoskeletal dynamics, metabolic process, and neurotransmission being related to p‐Tau. This workflow is useful as a complementary approach, for rapidly identifying aggregate components in multiple neurodegenerative diseases. It may also be used to evaluate the heterogeneity of Tau pathology that exists between patients and between diseases characterized by Tauopathies, a feature that can only be accurately evaluated using the post‐mortem tissue from patients.

## AUTHOR CONTRIBUTIONS

Conceptualization, SLR and RAWR; methodology, SLR, RAWR, VA, TZ, JW; biotinylation of samples and imaging, RAWR; preparation of samples for mass spectrometry, SLR; data acquisition using mass spectrometer, FC; data analysis, SLR and RAWR; writing—original draft preparation, SLR and RAWR; writing—review and editing, SR, RAWR, PS, MM, DLP, AL, RC; supervision, AL and RC.; All authors have read and agreed to the published version of the manuscript.

## FUNDING INFORMATION

This research has been supported by research grants from the National Health & Medical Research Council (APP1095215), Motor Neurone Disease Research Institute of Australia (IG1910, IG2221, IG2308), FightMND Drug Development Grant (04_DDG_2020), Stichting ALS (AV20200003), Australian Research Council (DP210103469) and philanthropic donations to the Macquarie University Centre for MND Research.

## CONFLICT OF INTEREST STATEMENT

The authors declare no conflict of interest.

## INSTITUTIONAL REVIEW BOARD STATEMENT: ETHICS APPROVAL AND CONSENT TO PARTICIPATE

Griffith University MSC/16/11/HREC, Macquarie University (HREA 5201600387).

## INFORMED CONSENT STATEMENT

Informed consent was obtained from all subjects involved in the study.

## Supporting information


Figure S1.

Figure S2.

Table S1.

Data S1.

## Data Availability

The mass spectrometry data can be found on the ProteomeXchange Consortium via the PRIDE partner repository (Perez‐Riverol, Csordas, et al. 2019). The dataset file identifier is PXD028237. PhosphoSite data can be accessed with the following. Project accession: PXD028770. Project DOI: Not applicable.

## References

[jnc15796-bib-0001] Ahmed, Z. , Asi, Y. T. , Lees, A. J. , Revesz, T. , & Holton, J. L. (2013). Identification and quantification of oligodendrocyte precursor cells in multiple system atrophy, progressive supranuclear palsy and parkinson's disease. Brain Pathology, 23(3), 263–273. 10.1111/j.1750-3639.2012.00637.x 22994884 PMC8029493

[jnc15796-bib-0002] Allen, M. , Wang, X. , Serie, D. J. , Strickland, S. L. , Burgess, J. D. , Koga, S. , Younkin, C. S. , Nguyen, T. T. , Malphrus, K. G. , Lincoln, S. J. , Alamprese, M. , Zhu, K. , Chang, R. , Carrasquillo, M. M. , Kouri, N. , Murray, M. E. , Reddy, J. S. , Funk, C. , Price, N. D. , … Ertekin‐Taner, N. (2018). Divergent brain gene expression patterns associate with distinct cell‐specific tau neuropathology traits in progressive supranuclear palsy. Acta Neuropathologica, 136(5), 709–727. 10.1007/s00401-018-1900-5 30136084 PMC6208732

[jnc15796-bib-0003] Alonso, A. D. C. , Grundke‐Iqbal, I. , Barra, H. S. , & Iqbal, K. (1997). Abnormal phosphorylation of tau and the mechanism of Alzheimer neurofibrillary degeneration: Sequestration of microtubule‐associated proteins 1 and 2 and the disassembly of microtubules by the abnormal tau. Proceedings of the National Academy of Sciences, 94(1), 298–303. 10.1073/pnas.94.1.298 PMC19321 8990203

[jnc15796-bib-0004] Ando, K. , Ndjim, M. , Turbant, S. , Fontaine, G. , Pregoni, G. , Dauphinot, L. , Yilmaz, Z. , Suain, V. , Mansour, S. , Authelet, M. , De Dekker, R. , Leroy, K. , Delatour, B. , Letournel, F. , Martin‐Négrier, M.‐L. , Chapon, F. , Godfraind, C. , Maurage, C.‐A. , Deramecourt, V. , … Brain Bank Neuro, C. E. B. N. N . (2020). The lipid phosphatase Synaptojanin 1 undergoes a significant alteration in expression and solubility and is associated with brain lesions in Alzheimer's disease. Acta Neuropathologica Communications, 8(1), 79. 10.1186/s40478-020-00954-1 32493451 PMC7268631

[jnc15796-bib-0005] Arakhamia, T. , Lee, C. E. , Carlomagno, Y. , Duong, D. M. , Kundinger, S. R. , Wang, K. , Williams, D. , DeTure, M. , Dickson, D. W. , Cook, C. N. , Seyfried, N. T. , Petrucelli, L. , & Fitzpatrick, A. W. P. (2020). Posttranslational Modifications Mediate the Structural Diversity of Tauopathy Strains. Cell, 180(4), 633–644.e612. 10.1016/j.cell.2020.01.027 32032505 PMC7491959

[jnc15796-bib-0006] Arendt, T. , Stieler, J. T. , & Holzer, M. (2016). Tau and tauopathies. Brain Research Bulletin, 126, 238–292. 10.1016/j.brainresbull.2016.08.018 27615390

[jnc15796-bib-0008] Ayyadevara, S. , Balasubramaniam, M. , Parcon, P. A. , Barger, S. W. , Griffin, W. S. T. , Alla, R. , Tackett, A. J. , Mackintosh, S. G. , Petricoin, E. , Zhou, W. , & Shmookler Reis, R. J. (2016). Proteins that mediate protein aggregation and cytotoxicity distinguish Alzheimer's hippocampus from normal controls. Aging Cell, 15(5), 924–939. 10.1111/acel.12501 27448508 PMC5013017

[jnc15796-bib-0009] Bakken, T. E. , Jorstad, N. L. , Hu, Q. , Lake, B. B. , Tian, W. , Kalmbach, B. E. , Crow, M. , Hodge, R. D. , Krienen, F. M. , Sorensen, S. A. , Eggermont, J. , Yao, Z. , Aevermann, B. D. , Aldridge, A. I. , Bartlett, A. , Bertagnolli, D. , Casper, T. , Castanon, R. G. , Crichton, K. , … Lein, E. S. (2020). Evolution of cellular diversity in primary motor cortex of human, marmoset monkey, and mouse. bioRxiv 2020.2003.2031.016972. 10.1038/s41586-021-03465-8

[jnc15796-bib-0010] Bar, D. Z. , Atkatsh, K. , Tavarez, U. , Erdos, M. R. , Gruenbaum, Y. , & Collins, F. S. (2018). Biotinylation by antibody recognition—a method for proximity labeling. Nature Methods, 15(2), 127–133. 10.1038/nmeth.4533 29256494 PMC5790613

[jnc15796-bib-0011] Blair, J. A. , Wang, C. , Hernandez, D. , Siedlak, S. L. , Rodgers, M. S. , Achar, R. K. , Fahmy, L. M. , Torres, S. L. , Petersen, R. B. , Zhu, X. , Casadesus, G. , & Lee, H. G. (2016). Individual case analysis of postmortem interval time on brain tissue preservation. PLoS One, 11(3), e0151615. 10.1371/journal.pone.0151615 26982086 PMC4794172

[jnc15796-bib-0012] Carrettiero, D. C. , Hernandez, I. , Neveu, P. , Papagiannakopoulos, T. , & Kosik, K. S. (2009). The cochaperone BAG2 sweeps paired helical filament‐ insoluble tau from the microtubule. The Journal of Neuroscience, 29(7), 2151. 10.1523/JNEUROSCI.4660-08.2009 19228967 PMC2768429

[jnc15796-bib-0013] Chen, Z. , Chen, J. A. , Shatunov, A. , Jones, A. R. , Kravitz, S. N. , Huang, A. Y. , Lawrence, L. , Lowe, J. K. , Lewis, C. M. , Payan, C. A. M. , Lieb, W. , Franke, A. , Deloukas, P. , Amouyel, P. , Tzourio, C. , Dartigues, J.‐F. , NNIPPS, Groups, B. S , Ludolph, A. , … Al‐Chalabi, A. (2019). Genome‐wide survey of copy number variants finds MAPT duplications in progressive supranuclear palsy. Movement Disorders, 34(7), 1049–1059. 10.1002/mds.27702 31059154

[jnc15796-bib-0014] Darwich, N. F. , Phan, J. M. , Kim, B. , Suh, E. , Papatriantafyllou, J. D. , Changolkar, L. , Nguyen, A. T. , O'Rourke, C. M. , He, Z. , Porta, S. , Gibbons, G. S. , Luk, K. C. , Papageorgiou, S. G. , Grossman, M. , Massimo, L. , Irwin, D. J. , McMillan, C. T. , Nasrallah, I. M. , Toro, C. , … Lee, E. B. (2020). Autosomal dominant VCP hypomorph mutation impairs disaggregation of PHF‐tau. Science, 370(6519), eaay8826. 10.1126/science.aay8826 33004675 PMC7818661

[jnc15796-bib-0015] Dou, F. , Netzer, W. J. , Tanemura, K. , Li, F. , Hartl, F. U. , Takashima, A. , Gouras, G. K. , Greengard, P. , & Xu, H. (2003). Chaperones increase association of tau protein with microtubules. Proceedings of the National Academy of Sciences, 100(2), 721–726. 10.1073/pnas.242720499 PMC141063 12522269

[jnc15796-bib-0016] Drummond, E. , Pires, G. , MacMurray, C. , Askenazi, M. , Nayak, S. , Bourdon, M. , Safar, J. , Ueberheide, B. , & Wisniewski, T. (2020). Phosphorylated tau interactome in the human Alzheimer's disease brain. Brain, 143(9), 2803–2817. 10.1093/brain/awaa223 32812023 PMC7526722

[jnc15796-bib-0017] Drummond, E. S. , Nayak, S. , Ueberheide, B. , & Wisniewski, T. (2015). Proteomic analysis of neurons microdissected from formalin‐fixed, paraffin‐embedded Alzheimer's disease brain tissue. Scientific Reports, 5, 15456. 10.1038/srep15456 26487484 PMC4614382

[jnc15796-bib-0018] Duraiyan, J. , Govindarajan, R. , Kaliyappan, K. , & Palanisamy, M. (2012). Applications of immunohistochemistry. Journal of Pharmacy And Bioallied Sciences, 4(6), 307–309. 10.4103/0975-7406.100281 23066277 PMC3467869

[jnc15796-bib-0019] Ebrahim, A. S. , Kulathingal, J. , Murray, M. E. , Casey‐Castanedes, M. , Dickson, D. W. , Yen, S.‐H. , & Sevlever, D. (2011). A proteomic study identifies different levels of light chain ferritin in corticobasal degeneration and progressive supranuclear palsy. Acta Neuropathologica, 122(6), 727–736. 10.1007/s00401-011-0888-x 22012136

[jnc15796-bib-0020] Ekblom, J. , Jossan, S. S. , Bergstrüm, M. , Oreland, L. , Walum, E. , & Aquilonius, S.‐M. (1993). Monoamine oxidase‐B in astrocytes. Glia, 8(2), 122–132. 10.1002/glia.440080208 8406673

[jnc15796-bib-0021] Falcon, B. , Zhang, W. , Murzin, A. G. , Murshudov, G. , Garringer, H. J. , Vidal, R. , Crowther, R. A. , Ghetti, B. , Scheres, S. H. W. , & Goedert, M. (2018). Structures of filaments from Pick's disease reveal a novel tau protein fold. Nature, 561(7721), 137–140. 10.1038/s41586-018-0454-y 30158706 PMC6204212

[jnc15796-bib-0022] Falcon, B. , Zhang, W. , Schweighauser, M. , Murzin, A. G. , Vidal, R. , Garringer, H. J. , Ghetti, B. , Scheres, S. H. W. , & Goedert, M. (2018). Tau filaments from multiple cases of sporadic and inherited Alzheimer's disease adopt a common fold. Acta Neuropathologica, 136(5), 699–708. 10.1007/s00401-018-1914-z 30276465 PMC6208733

[jnc15796-bib-0023] Falcon, B. , Zivanov, J. , Zhang, W. , Murzin, A. G. , Garringer, H. J. , Vidal, R. , Crowther, R. A. , Newell, K. L. , Ghetti, B. , Goedert, M. , & Scheres, S. H. W. (2019). Novel tau filament fold in chronic traumatic encephalopathy encloses hydrophobic molecules. Nature, 568(7752), 420–423. 10.1038/s41586-019-1026-5 30894745 PMC6472968

[jnc15796-bib-0024] Forrest, S. L. , Kril, J. J. , Stevens, C. H. , Kwok, J. B. , Hallupp, M. , Kim, W. S. , Huang, Y. , McGinley, C. V. , Werka, H. , Kiernan, M. C. , Götz, J. , Spillantini, M. G. , Hodges, J. R. , Ittner, L. M. , & Halliday, G. M. (2018). Retiring the term FTDP‐17 as MAPT mutations are genetic forms of sporadic frontotemporal tauopathies. Brain, 141(2), 521–534. 10.1093/brain/awx328 29253099 PMC5888940

[jnc15796-bib-0025] Fujioka, S. , Sanchez Contreras, M. Y. , Strongosky, A. J. , Ogaki, K. , Whaley, N. R. , Tacik, P. M. , van Gerpen, J. A. , Uitti, R. J. , Ross, O. A. , Wszolek, Z. K. , Rademakers, R. , & Dickson, D. W. (2015). Three sib‐pairs of autopsy‐confirmed progressive supranuclear palsy. Parkinsonism & related disorders, 21(2), 101–105. 10.1016/j.parkreldis.2014.10.028 25443551 PMC4306617

[jnc15796-bib-0026] Galili, T. , O'Callaghan, A. , Sidi, J. , & Sievert, C. (2018). heatmaply: an R package for creating interactive cluster heatmaps for online publishing. Bioinformatics, 34(9), 1600–1602. 10.1093/bioinformatics/btx657 29069305 PMC5925766

[jnc15796-bib-0027] Goedert, M. , Jakes, R. , & Vanmechelen, E. (1995). Monoclonal antibody AT8 recognises tau protein phosphorylated at both serine 202 and threonine 205. Neuroscience Letters, 189(3), 167–170. 10.1016/0304-3940(95)11484-E 7624036

[jnc15796-bib-0028] Haenig, C. , Atias, N. , Taylor, A. K. , Mazza, A. , Schaefer, M. H. , Russ, J. , Riechers, S.‐P. , Jain, S. , Coughlin, M. , Fontaine, J.‐F. , Freibaum, B. D. , Brusendorf, L. , Zenkner, M. , Porras, P. , Stroedicke, M. , Schnoegl, S. , Arnsburg, K. , Boeddrich, A. , Pigazzini, L. , … Wanker, E. E. (2020). Interactome mapping provides a network of neurodegenerative disease proteins and uncovers widespread protein aggregation in affected brains. Cell Reports, 32(7), 108050. 10.1016/j.celrep.2020.108050 32814053

[jnc15796-bib-0029] Hales, C. M. , Dammer, E. B. , Deng, Q. , Duong, D. M. , Gearing, M. , Troncoso, J. C. , Thambisetty, M. , Lah, J. J. , Shulman, J. M. , Levey, A. I. , & Seyfried, N. T. (2016). Changes in the detergent‐insoluble brain proteome linked to amyloid and tau in Alzheimer's Disease progression. PROTEOMICS, 16(23), 3042–3053. 10.1002/pmic.201600057 27718298 PMC5462625

[jnc15796-bib-0030] Hardies, K. , Cai, Y. , Jardel, C. , Jansen, A. C. , Cao, M. , May, P. , Djémié, T. , Hachon Le Camus, C. , Keymolen, K. , Deconinck, T. , Bhambhani, V. , Long, C. , Sajan, S. A. , Helbig, K. L. , Consortium, A. R. w. g. o. t. E. R , Suls, A. , Balling, R. , Helbig, I. , De Jonghe, P. , … Zara, F. (2016). Loss of SYNJ1 dual phosphatase activity leads to early onset refractory seizures and progressive neurological decline. Brain, 139(9), 2420–2430. 10.1093/brain/aww180 27435091 PMC4995362

[jnc15796-bib-0031] Hashiguchi, M. , Sobue, K. , & Paudel, H. K. (2000). 14‐3‐3ζ is an effector of tau protein phosphorylation. Journal of Biological Chemistry, 275(33), 25247–25254. 10.1074/jbc.M003738200 10840038

[jnc15796-bib-0032] Hedl, T. J. , San Gil, R. , Cheng, F. , Rayner, S. L. , Davidson, J. M. , De Luca, A. , Villalva, M. D. , Ecroyd, H. , Walker, A. K. , & Lee, A. (2019). Proteomics approaches for biomarker and drug target discovery in ALS and FTD. Frontiers in Neuroscience, 13(548). 10.3389/fnins.2019.00548 PMC6579929 31244593

[jnc15796-bib-0033] Höglinger, G. U. , Melhem, N. M. , Dickson, D. W. , Sleiman, P. M. A. , Wang, L.‐S. , Klei, L. , Rademakers, R. , de Silva, R. , Litvan, I. , Riley, D. E. , van Swieten, J. C. , Heutink, P. , Wszolek, Z. K. , Uitti, R. J. , Vandrovcova, J. , Hurtig, H. I. , Gross, R. G. , Maetzler, W. , Goldwurm, S. , … Schellenberg, G. D. (2011). Identification of common variants influencing risk of the tauopathy progressive supranuclear palsy. Nature Genetics, 43, 699. 10.1038/ng.859 21685912 PMC3125476

[jnc15796-bib-0034] Höglinger, G. U. , Respondek, G. , Stamelou, M. , Kurz, C. , Josephs, K. A. , Lang, A. E. , Mollenhauer, B. , Müller, U. , Nilsson, C. , Whitwell, J. L. , Arzberger, T. , Englund, E. , Gelpi, E. , Giese, A. , Irwin, D. J. , Meissner, W. G. , Pantelyat, A. , Rajput, A. , van Swieten, J. C. , … for the Movement Disorder Society–endorsed, P. S. P. S. G . (2017). Clinical diagnosis of progressive supranuclear palsy: The movement disorder society criteria. Movement Disorders, 32(6), 853–864. 10.1002/mds.26987 28467028 PMC5516529

[jnc15796-bib-0035] Hsieh, Y.‐C. , Guo, C. , Yalamanchili, H. K. , Abreha, M. , Al‐Ouran, R. , Li, Y. , Dammer, E. B. , Lah, J. J. , Levey, A. I. , Bennett, D. A. , De Jager, P. L. , Seyfried, N. T. , Liu, Z. , & Shulman, J. M. (2019). Tau‐mediated disruption of the spliceosome triggers cryptic RNA Splicing and Neurodegeneration in Alzheimer Disease. Cell Reports, 29(2), 301–316.e310. 10.1016/j.celrep.2019.08.104 31597093 PMC6919331

[jnc15796-bib-0036] Hung, V. , Udeshi, N. D. , Lam, S. S. , Loh, K. H. , Cox, K. J. , Pedram, K. , Carr, S. A. , & Ting, A. Y. (2016). Spatially resolved proteomic mapping in living cells with the engineered peroxidase APEX2. Nature Protocols, 11(3), 456–475. 10.1038/nprot.2016.018 26866790 PMC4863649

[jnc15796-bib-0037] Im, S. Y. , Kim, Y. E. , & Kim, Y. J. (2015). Genetics of progressive supranuclear palsy. Journal of Movement Disorders, 8(3), 122–129. 10.14802/jmd.15033 26413239 PMC4572662

[jnc15796-bib-0038] Jaesun, C. , Taegun, K. , Eun Jeoung, L. , Chang Hyun, K. , Yeon Soo, H. , Soon‐Kwang, H. , Sounghee, H. , & Sang Sun, K. (2004). 14‐3‐3 protein mediates phosphorylation of microtubule‐associated protein tau by serum‐ and glucocorticoid‐induced protein kinase 1. Molecules and Cells, 18(3), 360–368. Retrieved from. http://www.molcells.org/journal/view.html?doi 15650334

[jnc15796-bib-0039] Julien, C. , Bretteville, A. , & Planel, E. (2012). Biochemical isolation of insoluble tau in transgenic mouse models of tauopathies. In E. M. Sigurdsson , M. Calero , & M. Gasset (Eds.), Amyloid Proteins: Methods and Protocols (pp. 473–491). Humana Press. 10.1007/978-1-61779-551-0_32 22528110

[jnc15796-bib-0040] Kaneko, Y. , Kitamoto, T. , Tateishi, J. , & Yamaguchi, K. (1989). Ferritin immunohistochemistry as a marker for microglia. Acta Neuropathologica, 79(2), 129–136. 10.1007/BF00294369 2596262

[jnc15796-bib-0041] Kavanagh, T. , Halder, A. , & Drummond, E. (2022). Tau interactome and RNA binding proteins in neurodegenerative diseases. Mol Neurodegener, 17(1), 66. 10.1186/s13024-022-00572-6 36253823 PMC9575286

[jnc15796-bib-0042] Kepchia, D. , Huang, L. , Dargusch, R. , Rissman, R. A. , Shokhirev, M. N. , Fischer, W. , & Schubert, D. (2020). Diverse proteins aggregate in mild cognitive impairment and Alzheimer's disease brain. Alzheimer's Research & Therapy, 12(1), 75. 10.1186/s13195-020-00641-2 PMC7305608 32560738

[jnc15796-bib-0043] Killinger, B. A. , Marshall, L. L. , Chatterjee, D. , Chu, Y. , Bras, J. , Guerreiro, R. , & Kordower, J. H. (2022). In situ proximity labeling identifies Lewy pathology molecular interactions in the human brain. Proceedings of the National Academy of Sciences of the United States of America, 119(5), e2114405119. 10.1073/pnas.2114405119 35082147 PMC8812572

[jnc15796-bib-0044] Kovacs, G. G. (2015). Invited review: Neuropathology of tauopathies: principles and practice. Neuropathology and Applied Neurobiology, 41(1), 3–23. 10.1111/nan.12208 25495175

[jnc15796-bib-0045] Kovacs, G. G. , Lukic, M. J. , Irwin, D. J. , Arzberger, T. , Respondek, G. , Lee, E. B. , Coughlin, D. , Giese, A. , Grossman, M. , Kurz, C. , McMillan, C. T. , Gelpi, E. , Compta, Y. , van Swieten, J. C. , Laat, L. D. , Troakes, C. , Al‐Sarraj, S. , Robinson, J. L. , Roeber, S. , … Höglinger, G. U. (2020). Distribution patterns of tau pathology in progressive supranuclear palsy. Acta Neuropathologica, 140(2), 99–119. 10.1007/s00401-020-02158-2 32383020 PMC7360645

[jnc15796-bib-0046] Lake Blue, B. , Ai, R. , Kaeser Gwendolyn, E. , Salathia Neeraj, S. , Yung Yun, C. , Liu, R. , Wildberg, A. , Gao, D. , Fung, H.‐L. , Chen, S. , Vijayaraghavan, R. , Wong, J. , Chen, A. , Sheng, X. , Kaper, F. , Shen, R. , Ronaghi, M. , Fan, J.‐B. , Wang, W. , … Zhang, K. (2016). Neuronal subtypes and diversity revealed by single‐nucleus RNA sequencing of the human brain. Science, 352(6293), 1586–1590. 10.1126/science.aaf1204 27339989 PMC5038589

[jnc15796-bib-0047] Lê, S. , Josse, J. , & Husson, F. (2008). FactoMineR: An R Package for Multivariate Analysis. Journal of Statistical Software, 25(1), 1–18. 10.18637/jss.v025.i01

[jnc15796-bib-0048] Lester, E. , Ooi, F. K. , Bakkar, N. , Ayers, J. , Woerman, A. L. , Wheeler, J. , Bowser, R. , Carlson, G. A. , Prusiner, S. B. , & Parker, R. (2021). Tau aggregates are RNA‐protein assemblies that mislocalize multiple nuclear speckle components. Neuron, 109(10), 1675–1691.e1679. 10.1016/j.neuron.2021.03.026 33848474 PMC8141031

[jnc15796-bib-0049] Li, T. , & Paudel, H. K. (2016). 14‐3‐3ζ mediates tau aggregation in human neuroblastoma M17 Cells. PLoS One, 11(8), e0160635. 10.1371/journal.pone.0160635 27548710 PMC4993442

[jnc15796-bib-0050] Liu, C. , Song, X. , Nisbet, R. , & Götz, J. (2016). Co‐immunoprecipitation with tau isoform‐specific antibodies reveals distinct protein interactions and highlights a putative role for 2n tau in disease*. Journal of Biological Chemistry, 291(15), 8173–8188. 10.1074/jbc.M115.641902 26861879 PMC4825019

[jnc15796-bib-0051] Lobingier, B. T. , Hüttenhain, R. , Eichel, K. , Miller, K. B. , Ting, A. Y. , von Zastrow, M. , & Krogan, N. J. (2017). An approach to spatiotemporally resolve protein interaction networks in living cells. Cell, 169(2), 350–360.e312. 10.1016/j.cell.2017.03.022 28388416 PMC5616215

[jnc15796-bib-0052] Magnani, E. , Fan, J. , Gasparini, L. , Golding, M. , Williams, M. , Schiavo, G. , Goedert, M. , Amos, L. A. , & Spillantini, M. G. (2007). Interaction of tau protein with the dynactin complex. The EMBO Journal, 26(21), 4546–4554. 10.1038/sj.emboj.7601878 17932487 PMC2063488

[jnc15796-bib-0053] Malia, T. J. , Teplyakov, A. , Ernst, R. , Wu, S.‐J. , Lacy, E. R. , Liu, X. , Vandermeeren, M. , Mercken, M. , Luo, J. , Sweet, R. W. , & Gilliland, G. L. (2016). Epitope mapping and structural basis for the recognition of phosphorylated tau by the anti‐tau antibody AT8. Proteins: Structure, Function, and Bioinformatics, 84(4), 427–434. 10.1002/prot.24988 PMC5067699 26800003

[jnc15796-bib-0054] Matthews, T. A. , & Johnson, G. V. W. (2005). 14‐3‐3ζ does not increase GSK3β‐mediated tau phosphorylation in cell culture models. Neuroscience Letters, 384(3), 211–216. 10.1016/j.neulet.2005.04.101 15963640

[jnc15796-bib-0055] Maziuk, B. F. , Apicco, D. J. , Cruz, A. L. , Jiang, L. , Ash, P. E. A. , da Rocha, E. L. , Zhang, C. , Yu, W. H. , Leszyk, J. , Abisambra, J. F. , Li, H. , & Wolozin, B. (2018). RNA binding proteins co‐localize with small tau inclusions in tauopathy. Acta Neuropathologica Communications, 6(1), 71. 10.1186/s40478-018-0574-5 30068389 PMC6069705

[jnc15796-bib-0056] McInnes, J. , Wierda, K. , Snellinx, A. , Bounti, L. , Wang, Y.‐C. , Stancu, I.‐C. , Apóstolo, N. , Gevaert, K. , Dewachter, I. , Spires‐Jones, T. L. , De Strooper, B. , De Wit, J. , Zhou, L. , & Verstreken, P. (2018). Synaptogyrin‐3 Mediates Presynaptic Dysfunction Induced by Tau. Neuron, 97(4), 823–835.e828. 10.1016/j.neuron.2018.01.022 29398363

[jnc15796-bib-0057] Meier, S. , Bell, M. , Lyons, D. N. , Ingram, A. , Chen, J. , Gensel, J. C. , Zhu, H. , Nelson, P. T. , & Abisambra, J. F. (2015). Identification of novel tau interactions with endoplasmic reticulum proteins in Alzheimer's disease brain. Journal of Alzheimer's Disease, 48, 687–702. 10.3233/JAD-150298 PMC4881838 26402096

[jnc15796-bib-0058] Muchowski, P. J. , & Wacker, J. L. (2005). Modulation of neurodegeneration by molecular chaperones. Nature Reviews Neuroscience, 6(1), 11–22. 10.1038/nrn1587 15611723

[jnc15796-bib-0059] Nellist, M. , Goedbloed, M. A. , de Winter, C. , Verhaaf, B. , Jankie, A. , Reuser, A. J. J. , van den Ouweland, A. M. W. , van der Sluijs, P. , & Halley, D. J. J. (2002). Identification and Characterization of the Interaction between Tuberin and 14‐3‐3ζ*. Journal of Biological Chemistry, 277(42), 39417–39424. 10.1074/jbc.M204802200 12176984

[jnc15796-bib-0060] Nuebling, G. , Hensler, M. , Paul, S. , Zwergal, A. , Crispin, A. , & Lorenzl, S. (2016). PROSPERA: a randomized, controlled trial evaluating rasagiline in progressive supranuclear palsy. Journal of Neurology, 263(8), 1565–1574. 10.1007/s00415-016-8169-1 27230855

[jnc15796-bib-0061] Orr, M. E. , Sullivan, A. C. , & Frost, B. (2017). A brief overview of tauopathy: Causes, consequences, and therapeutic strategies. Trends in Pharmacological Sciences, 38(7), 637–648. 10.1016/j.tips.2017.03.011 28455089 PMC5476494

[jnc15796-bib-0062] Pace, M. C. , Xu, G. , Fromholt, S. , Howard, J. , Crosby, K. , Giasson, B. I. , Lewis, J. , & Borchelt, D. R. (2018). Changes in proteome solubility indicate widespread proteostatic disruption in mouse models of neurodegenerative disease. Acta Neuropathologica, 136(6), 919–938. 10.1007/s00401-018-1895-y 30140941 PMC6411038

[jnc15796-bib-0063] Papanikolopoulou, K. , Grammenoudi, S. , Samiotaki, M. , & Skoulakis, E. M. C. (2018). Differential effects of 14‐3‐3 dimers on Tau phosphorylation, stability and toxicity in vivo. Human Molecular Genetics, 27(13), 2244–2261. 10.1093/hmg/ddy129 29659825

[jnc15796-bib-0064] Pastor, P. , Ezquerra, M. , Perez, J. C. , Chakraverty, S. , Norton, J. , Racette, B. A. , McKeel, D. , Perlmutter, J. S. , Tolosa, E. , & Goate, A. M. (2004). Novel haplotypes in 17q21 are associated with progressive supranuclear palsy. Annals of Neurology, 56(2), 249–258. 10.1002/ana.20178 15293277

[jnc15796-bib-0065] Pikkarainen, M. , Martikainen, P. , & Alafuzoff, I. (2010). The effect of prolonged fixation time on immunohistochemical staining of common neurodegenerative disease markers. Journal of Neuropathology & Experimental Neurology, 69(1), 40–52. 10.1097/NEN.0b013e3181c6c13d 20010304

[jnc15796-bib-0066] Pires, G. , McElligott, S. , Drusinsky, S. , Halliday, G. , Potier, M.‐C. , Wisniewski, T. , & Drummond, E. (2019). Secernin‐1 is a novel phosphorylated tau binding protein that accumulates in Alzheimer's disease and not in other tauopathies. Acta Neuropathologica Communications, 7(1), 195. 10.1186/s40478-019-0848-6 31796108 PMC6892024

[jnc15796-bib-0067] Prikas, E. , Paric, E. , Asih, P. R. , Stefanoska, K. , Stefen, H. , Fath, T. , Poljak, A. , & Ittner, A. (2022). Tau target identification reveals NSF‐dependent effects on AMPA receptor trafficking and memory formation. The EMBO Journal, 41(18), e10242. 10.15252/embj.2021110242 35993331 PMC9475529

[jnc15796-bib-0068] Qureshi, H. Y. , Li, T. , MacDonald, R. , Cho, C. M. , Leclerc, N. , & Paudel, H. K. (2013). Interaction of 14‐3‐3ζ with Microtubule‐Associated Protein Tau within Alzheimer's Disease Neurofibrillary Tangles. Biochemistry, 52(37), 6445–6455. 10.1021/bi400442d 23962087

[jnc15796-bib-0069] Ramos‐Vara, J. A. (2005). Technical Aspects of Immunohistochemistry. Veterinary Pathology, 42(4), 405–426. 10.1354/vp.42-4-405 16006601

[jnc15796-bib-0070] Rizo, J. , & Xu, J. (2015). The synaptic vesicle release machinery. Annual Review of Biophysics, 44(1), 339–367. 10.1146/annurev-biophys-060414-034057 26098518

[jnc15796-bib-0071] Roux, K. J. , Kim, D. I. , Raida, M. , & Burke, B. (2012). A promiscuous biotin ligase fusion protein identifies proximal and interacting proteins in mammalian cells. Journal of Cell Biology, 196(6), 801–810. 10.1083/jcb.201112098 22412018 PMC3308701

[jnc15796-bib-0072] Sadik, G. , Tanaka, T. , Kato, K. , Yamamori, H. , Nessa, B. N. , Morihara, T. , & Takeda, M. (2009). Phosphorylation of tau at Ser214 mediates its interaction with 14‐3‐3 protein: implications for the mechanism of tau aggregation. Journal of Neurochemistry, 108(1), 33–43. 10.1111/j.1471-4159.2008.05716.x 19014373

[jnc15796-bib-0073] Saliba, A.‐E. , Westermann, A. J. , Gorski, S. A. , & Vogel, J. (2014). Single‐cell RNA‐seq: advances and future challenges. Nucleic Acids Research, 42(14), 8845–8860. 10.1093/nar/gku555 25053837 PMC4132710

[jnc15796-bib-0074] Schneider, S. A. , & Alcalay, R. N. (2017). Neuropathology of genetic synucleinopathies with parkinsonism: Review of the literature. Movement Disorders, 32(11), 1504–1523. 10.1002/mds.27193 29124790 PMC5726430

[jnc15796-bib-0075] Shi, Y. , Zhang, W. , Yang, Y. , Murzin, A. G. , Falcon, B. , Kotecha, A. , van Beers, M. , Tarutani, A. , Kametani, F. , Garringer, H. J. , Vidal, R. , Hallinan, G. I. , Lashley, T. , Saito, Y. , Murayama, S. , Yoshida, M. , Tanaka, H. , Kakita, A. , Ikeuchi, T. , … Scheres, S. H. W. (2021). Structure‐based classification of tauopathies. Nature. 10.1038/s41586-021-03911-7 PMC7611841 34588692

[jnc15796-bib-0076] Sinsky, J. , Majerova, P. , Kovac, A. , Kotlyar, M. , Jurisica, I. , & Hanes, J. (2020). Physiological tau interactome in brain and its link to tauopathies. Journal of Proteome Research, 19(6), 2429–2442. 10.1021/acs.jproteome.0c00137 32357304

[jnc15796-bib-0077] Sluchanko, N. N. , Seit‐Nebi, A. S. , & Gusev, N. B. (2009). Effect of phosphorylation on interaction of human tau protein with 14‐3‐3ζ. Biochemical and Biophysical Research Communications, 379(4), 990–994. 10.1016/j.bbrc.2008.12.164 19138662

[jnc15796-bib-0078] Söderberg, O. , Leuchowius, K.‐J. , Gullberg, M. , Jarvius, M. , Weibrecht, I. , Larsson, L.‐G. , & Landegren, U. (2008). Characterizing proteins and their interactions in cells and tissues using the in situ proximity ligation assay. Methods, 45(3), 227–232. 10.1016/j.ymeth.2008.06.014 18620061

[jnc15796-bib-0079] Stutzbach, L. D. , Xie, S. X. , Naj, A. C. , Albin, R. , Gilman, S. , Lee, V. M. Y. , Trojanowski, J. Q. , Devlin, B. , & Schellenberg, G. D. (2013). The unfolded protein response is activated in disease‐affected brain regions in progressive supranuclear palsy and Alzheimer's disease. Acta Neuropathologica Communications, 1(1), 31. 10.1186/2051-5960-1-31 24252572 PMC3893579

[jnc15796-bib-0080] Taniguchi‐Watanabe, S. , Arai, T. , Kametani, F. , Nonaka, T. , Masuda‐Suzukake, M. , Tarutani, A. , Murayama, S. , Saito, Y. , Arima, K. , Yoshida, M. , Akiyama, H. , Robinson, A. , Mann, D. M. A. , Iwatsubo, T. , & Hasegawa, M. (2016). Biochemical classification of tauopathies by immunoblot, protein sequence and mass spectrometric analyses of sarkosyl‐insoluble and trypsin‐resistant tau. Acta Neuropathologica, 131(2), 267–280. 10.1007/s00401-015-1503-3 26538150 PMC4713716

[jnc15796-bib-0081] Thul Peter, J. , Åkesson, L. , Wiking, M. , Mahdessian, D. , Geladaki, A. , Ait Blal, H. , Alm, T. , Asplund, A. , Björk, L. , Breckels Lisa, M. , Bäckström, A. , Danielsson, F. , Fagerberg, L. , Fall, J. , Gatto, L. , Gnann, C. , Hober, S. , Hjelmare, M. , Johansson, F. , … Lundberg, E. (2017). A subcellular map of the human proteome. Science, 356(6340), eaal3321. 10.1126/science.aal3321 28495876

[jnc15796-bib-0082] Tong, J. , Ang, L.‐C. , Williams, B. , Furukawa, Y. , Fitzmaurice, P. , Guttman, M. , Boileau, I. , Hornykiewicz, O. , & Kish, S. J. (2015). Low levels of astroglial markers in Parkinson's disease: Relationship to α‐synuclein accumulation. Neurobiology of Disease, 82, 243–253. 10.1016/j.nbd.2015.06.010 26102022 PMC4641013

[jnc15796-bib-0083] Tong, J. , Rathitharan, G. , Meyer, J. H. , Furukawa, Y. , Ang, L.‐C. , Boileau, I. , Guttman, M. , Hornykiewicz, O. , & Kish, S. J. (2017). Brain monoamine oxidase B and A in human parkinsonian dopamine deficiency disorders. Brain, 140(9), 2460–2474. 10.1093/brain/awx172 29050386 PMC6669411

[jnc15796-bib-0084] Tugaeva, K. V. , Tsvetkov, P. O. , & Sluchanko, N. N. (2017). Bacterial co‐expression of human Tau protein with protein kinase A and 14‐3‐3 for studies of 14‐3‐3/phospho‐Tau interaction. PLoS One, 12(6), e0178933. 10.1371/journal.pone.0178933 28575131 PMC5456370

[jnc15796-bib-0085] Uhlén, M. , Fagerberg, L. , Hallström Björn, M. , Lindskog, C. , Oksvold, P. , Mardinoglu, A. , Sivertsson, Å. , Kampf, C. , Sjöstedt, E. , Asplund, A. , Olsson, I. , Edlund, K. , Lundberg, E. , Navani, S. , Szigyarto Cristina, A.‐K. , Odeberg, J. , Djureinovic, D. , Takanen Jenny, O. , Hober, S. , … Pontén, F. (2015). Tissue‐based map of the human proteome. Science, 347(6220), 1260419. 10.1126/science.1260419 25613900

[jnc15796-bib-0086] Wang, P. , Joberty, G. , Buist, A. , Vanoosthuyse, A. , Stancu, I.‐C. , Vasconcelos, B. , Pierrot, N. , Faelth‐Savitski, M. , Kienlen‐Campard, P. , Octave, J.‐N. , Bantscheff, M. , Drewes, G. , Moechars, D. , & Dewachter, I. (2017). Tau interactome mapping based identification of Otub1 as Tau deubiquitinase involved in accumulation of pathological Tau forms in vitro and in vivo. Acta Neuropathologica, 133(5), 731–749. 10.1007/s00401-016-1663-9 28083634 PMC5390007

[jnc15796-bib-0087] Williams, D. R. , Holton, J. L. , Strand, C. , Pittman, A. , de Silva, R. , Lees, A. J. , & Revesz, T. (2007). Pathological tau burden and distribution distinguishes progressive supranuclear palsy‐parkinsonism from Richardson's syndrome. Brain, 130(6), 1566–1576. 10.1093/brain/awm104 17525140

[jnc15796-bib-0088] Wray, S. , Saxton, M. , Anderton, B. H. , & Hanger, D. P. (2008). Direct analysis of tau from PSP brain identifies new phosphorylation sites and a major fragment of N‐terminally cleaved tau containing four microtubule‐binding repeats. Journal of Neurochemistry, 105(6), 2343–2352. 10.1111/j.1471-4159.2008.05321.x 18315566

[jnc15796-bib-0089] Yokoyama, J. S. , Karch, C. M. , Fan, C. C. , Bonham, L. W. , Kouri, N. , Ross, O. A. , Rademakers, R. , Kim, J. , Wang, Y. , Höglinger, G. U. , Müller, U. , Ferrari, R. , Hardy, J. , Momeni, P. , Sugrue, L. P. , Hess, C. P. , James Barkovich, A. , Boxer, A. L. , Seeley, W. W. , … International, F. T. D. G. C . (2017). Shared genetic risk between corticobasal degeneration, progressive supranuclear palsy, and frontotemporal dementia. Acta Neuropathologica, 133(5), 825–837. 10.1007/s00401-017-1693-y 28271184 PMC5429027

[jnc15796-bib-0090] Yuan, S. H. , Hiramatsu, N. , Liu, Q. , Sun, X. V. , Lenh, D. , Chan, P. , Chiang, K. , Koo, E. H. , Kao, A. W. , Litvan, I. , & Lin, J. H. (2018). Tauopathy‐associated PERK alleles are functional hypomorphs that increase neuronal vulnerability to ER stress. Human Molecular Genetics, 27(22), 3951–3963. 10.1093/hmg/ddy297 30137327 PMC6216228

[jnc15796-bib-0091] Yuan, Z. , Agarwal‐Mawal, A. , & Paudel, H. K. (2004). 14‐3‐3 binds to and mediates phosphorylation of microtubule‐associated tau protein by Ser9‐phosphorylated glycogen synthase kinase 3β in the brain*. Journal of Biological Chemistry, 279(25), 26105–26114. 10.1074/jbc.M308298200 15073173

[jnc15796-bib-0092] Zhang, W. , Tarutani, A. , Newell, K. L. , Murzin, A. G. , Matsubara, T. , Falcon, B. , Vidal, R. , Garringer, H. J. , Shi, Y. , Ikeuchi, T. , Murayama, S. , Ghetti, B. , Hasegawa, M. , Goedert, M. , & Scheres, S. H. W. (2020). Novel tau filament fold in corticobasal degeneration. Nature. 10.1038/s41586-020-2043-0 PMC7148158 32050258

[jnc15796-bib-0093] Zhang, Y. , Sloan, S. A. , Clarke, L. E. , Caneda, C. , Plaza, C. A. , Blumenthal, P. D. , Vogel, H. , Steinberg, G. K. , Edwards, M. S. B. , Li, G. , Duncan, J. A., III , Cheshier, S. H. , Shuer, L. M. , Chang, E. F. , Grant, G. A. , Gephart, M. G. H. , & Barres, B. A. (2016). Purification and characterization of progenitor and mature human astrocytes reveals transcriptional and functional differences with mouse. Neuron, 89(1), 37–53. 10.1016/j.neuron.2015.11.013 26687838 PMC4707064

