# Supplementary figures and images for: Identification of phosphorylated tau protein interactors in progressive supranuclear palsy (PSP) reveals networks involved in protein degradation, stress response, cytoskeletal dynamics, metabolic processes, and neurotransmission
Source: J Neurochem. 2023 Mar 21;165(4):563–86. doi: 10.1111/jnc.15796 (PMC10953353; doi:10.1111/jnc.15796)

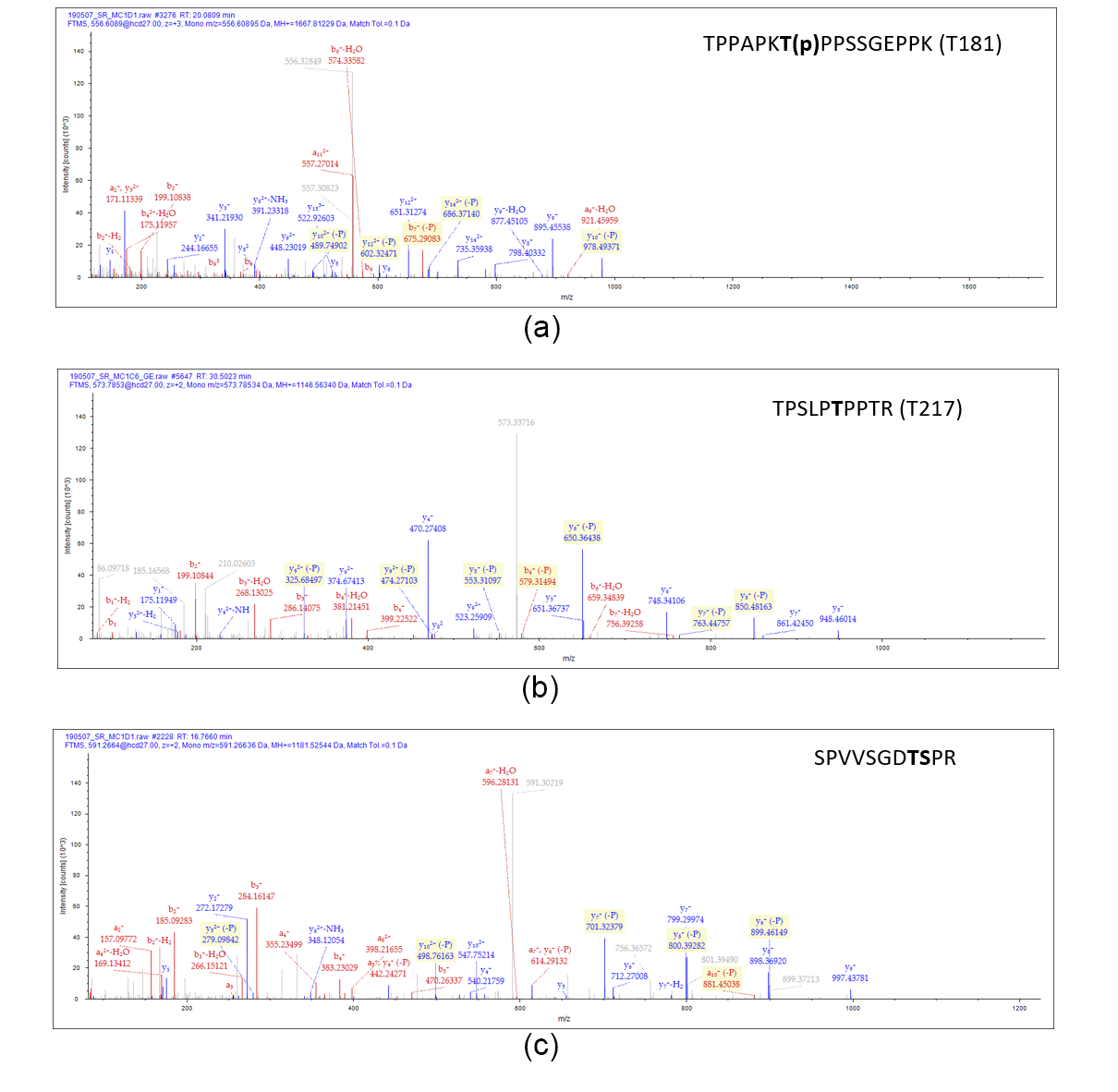

Supplement: Supplementary file 1 — Figure S1. [file JNC-165-563-s003.tif]

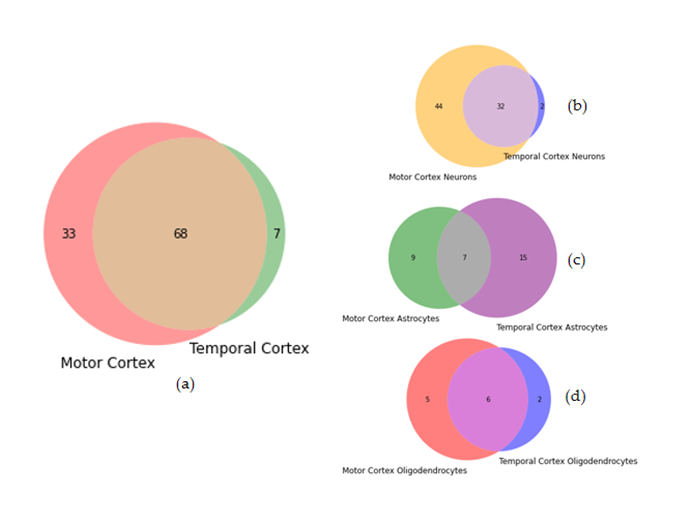

Supplement: Supplementary file 2 — Figure S2. [file JNC-165-563-s004.TIF]
