# Supplementary material for: Identification of phosphorylated tau protein interactors in progressive supranuclear palsy (PSP) reveals networks involved in protein degradation, stress response, cytoskeletal dynamics, metabolic processes, and neurotransmission
Source: J Neurochem. 2023 Mar 21;165(4):563–86. doi: 10.1111/jnc.15796 (PMC10953353; doi:10.1111/jnc.15796)
Supplement: Supplementary file 4 — Data S1. [file JNC-165-563-s001.docx]

**SUPPLEMENTS TO:**

**Identification of phosphorylated Tau protein interactors in progressive supranuclear palsy (PSP) reveals networks involved in protein degradation, stress response, cytoskeletal dynamics, metabolic processes, and neurotransmission.**

**Rowan A. W. Radford^1^**^†^*,**Stephanie L. Rayner^1^**^†^**, Paulina Szwaja^1^, Marco Morsch^1^, Flora Cheng^1^, Tianyi Zhu^2^, Jocelyn Widagdo ^2^, Victor Anggono^2^, Dean L. Pountney^3^, Roger Chung^1^, and Albert Lee^1*^**^,^

1. Macquarie University Centre for Motor Neuron Disease Research, Macquarie Medical School, Faculty of Medicine, Health, and Human Sciences, Macquarie University, North Ryde, NSW, Australia.

2. Clem Jones Centre for Ageing Dementia Research, Queensland Brain Institute, The University of Queensland, Brisbane, QLD, Australia

3. School of Pharmacy and Medical Sciences, Griffith University, Gold Coast, QLD, Australia.

* Corresponding authors: [rowan.radford@mq.edu.au](mailto:rowan.radford@mq.edu.au), albert.lee@mq.edu.au

^†^ These authors would like to be considered as co-first authors.

Present Address: Centre for Motor Neuron Disease Research, Department of Biomedical Sciences

Level 1, 75 Talavera Rd Macquarie University, North Ryde NSW, 2109 Australia


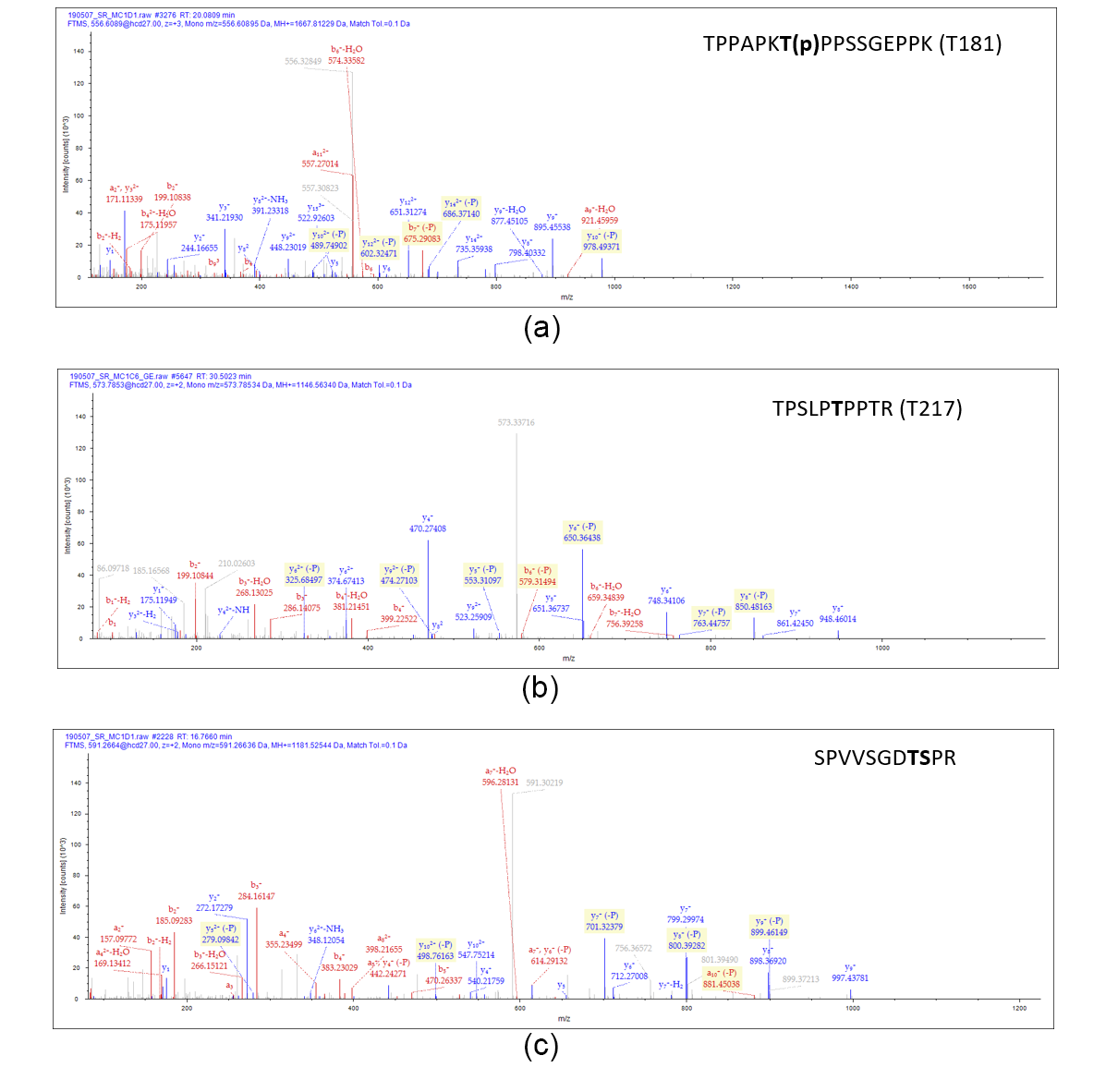


**Supplementary Figure 1.** Confidently identified phosphopeptides identified in PSP patients. (a) Phosphospectra for position T181. (b) Phosphospectra for position T217. (c) Phosphospectra for position T/S 403/404.


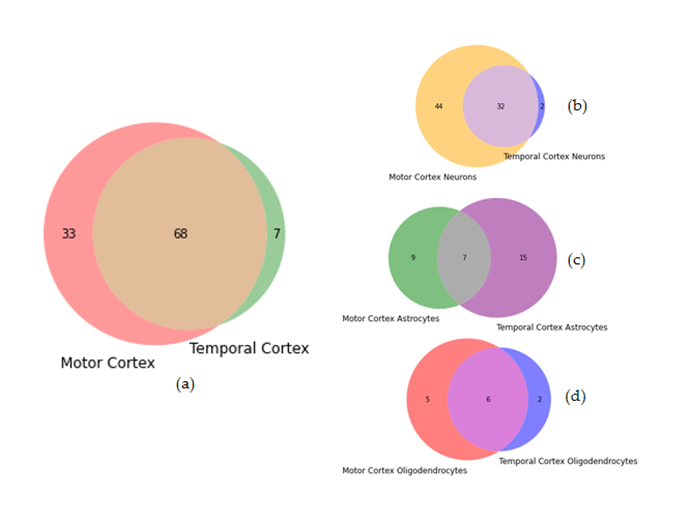


**Supplementary Figure 2.** Predicted gene enrichement in Motor and Temporal Cortices RNA-seq datasets. (a) Unique genes predicted to be enriched in cell types of the motor cortex shown in red. Genes predicted to have an enriched expression in an indicvidual cell type from temporal cortex RNA-seq dataset shown in green. Genes which have a predicted enrichement in both datasets is displayed in orange. (b) Genes enriched in neurons from the motor cortex dataset in orange, while unique genes enriched in neurons from the temporal crtex data set in blue. 32 genes were predicted to be enriched in neurons in both datasets shown in purple. (c) 7 genes are predicted to be enriched in astrocytes (grey) in both datasets, 15 genes are enriched only in temporal cortex (purple), while 9 are only enriched in the motor cortex dataset (green). (d) 6 genes have a predicted enriched expression in both datasets (purple), 5 only in the motor cortex dataset (red) and two unique to the temporal cortex dataset (blue).
